# Getting to intent: Are social norms influencing intentions to use modern contraception in the DRC?

**DOI:** 10.1371/journal.pone.0219617

**Published:** 2019-07-16

**Authors:** Elizabeth Costenbader, Seth Zissette, Andres Martinez, Katherine LeMasters, Nana Apenem Dagadu, Prabu Deepan, Bryan Shaw

**Affiliations:** 1 FHI 360, Durham, NC, United States of America; 2 University of North Carolina Gillings School of Public Health, Chapel Hill, NC, United States of America; 3 Institute of Reproductive Health at Georgetown University, Washington, DC, United States of America; 4 Tearfund, Teddington, United Kingdom; Monash University, AUSTRALIA

## Abstract

Meeting the reproductive health needs of women in post-conflict settings is a global health priority. In the Democratic Republic of the Congo, social norms perpetuate gender-based violence and contribute to low contraceptive use and high fertility. The Masculinité, Famille, et Foi (MFF) intervention is working with communities in Kinshasa to create normative environments supportive of modern contraception access and use. Our analysis uses survey data collected from 900 men and women in 17 community groups prior to the MFF intervention. We aimed to measure the extent to which social norms influence intentions to use modern contraception. Using multiple items to assess social norms and reference groups related to family planning and gender equity, we identified four distinct social norms constructs through factor analysis. Through structural equation modeling, we found that social norms influence intentions to use modern contraception overall, but that normative influence varies by gender.

## Introduction

Meeting the family planning reproductive health (FPRH) needs of women in post-conflict settings is a global health priority [1, 2]. The Democratic Republic of the Congo (DRC) is still recovering from protracted war that began in the aftermath of the 1994 Rwandan genocide and officially ended in 2003. During this time, sexual and gender-based violence (GBV) was widely used as a weapon of war [3–5]. Currently, in the DRC, violence still ensues in many regions of the country, and the rate of GBV is among the highest in the world per the DRC’s 2014 Demographic and Health Survey [6]. Among different forms of GBV, intimate partner violence (IPV) is associated with lower rates of contraceptive use [7]. Not surprisingly, in the DRC, rates of modern contraceptive use among all women remain very low at only 10.3% as of 2017 and unmet need remains high at 40.0% [6, 8].

Similarly, the history of conflict may have worsened gender inequitable beliefs among both men and women in the DRC [9]. Previous studies have found majorities of both men and women, but particularly men, in the DRC to agree with gender inequitable beliefs, such as those that a man should have the final say in family matters and the woman’s primary role is caring for her home [9–11]. These beliefs are paired with gender inequitable beliefs around sex. Men in the DRC have also reported beliefs that men need sex more than women do; that “men don’t talk about sex, they just do it”; and that it is the responsibility of women to avoid pregnancy [9–12].

Identifying and working to modify social factors which perpetuate gender inequities and GBV will be critical to achieving improvements in FPRH in the DRC. Specifically, social and gender norms—the shared understandings of how oneself and others should behave [13]—can significantly influence a woman’s ability to plan her pregnancies, as well as a couple’s ability to openly discuss and negotiate family planning (FP) use. The social norm that a man should make decisions for his household and is justified to use physical force to enforce his decisions is one such example. Currently, there are a growing number of interventions designed to counter such social norms [14]. However, to date, the evidence base upon which these interventions are being designed and evaluated is small, resulting largely from the fact that the measurement of social norms has lagged behind [15, 16]. In partnership with a team of global health experts—Georgetown’s Institute for Reproductive Health (IRH), FHI 360, Johns Hopkins Global Early Adolescent Study (GEAS), Population Services International (PSI), Save the Children, and Tearfund—the Passages Project is working to establish an evidence base on scalable social norm change approaches that reduce stigma and myths related to FP use, increase male engagement in FP, reduce sexual and GBV, and improve gender-equitable attitudes and behaviors.

The DRC is a predominantly Christian country where, similar to much of the developing world, the majority of the population attends religious services often, if not always [10, 17]. In such settings, FPRH programs have often found religious leaders to be important community leaders and therefore important allies and effective advocates for dispelling myths associated with use of FP methods [18–20]. Tearfund, a Passages partner, is a Christian relief and development agency that works locally in Kinshasa. Starting in 2014, Tearfund developed and collected evidence [21] to demonstrate the effectiveness of an intervention approach, known locally as the Transforming Masculinities (TM) approach, which engages religious leaders and faith communities to encourage reflection, discussion, and action to promote gender-equitable beliefs with the goal of reducing GBV. As part of the Passages project, partner organizations added components to the original TM curriculum approach that focus on use of FP methods. This new enhanced intervention, known locally as Masculinité, Famille, et Foi (MFF), is currently being implemented in eight Protestant congregations in Kinshasa and soon will be scaled up in an additional nine Protestant congregations in Kinshasa. The MFF intervention is based on the idea that religious leaders can help communities understand that FP does not go against their faith, as well as reduce the community’s social acceptance of GBV and other gender inequalities, which would, in turn, create normative environments that are more supportive of women and men’s access to and use of modern FP. The MFF intervention is designed to work with newly married couples (NMCs) and first-time parents (FTPs), both because they are within the age range of people most in need of FP methods and because previous research has shown that working with individuals in transitional life stages, such as getting married and starting a family, presents an opportunity to intervene at a time when identities, norms, and behaviors are changing [22].

In this paper, we are interested in assessing the normative environment for FP use in the MFF congregations prior to implementation of the intervention. We sought evidence for norms directly related to FP, as well as those related to gender equity, that theoretically, we expect to indirectly affect couples’ use of contraception. We also assessed which groups of people had the most influence on participants’ thinking about contraceptive use. We then examined the association between social norm constructs and intention to use a modern contraceptive, as modern contraceptive use was the ultimate goal of the intervention.

### Conceptual model

**Fig 1:**
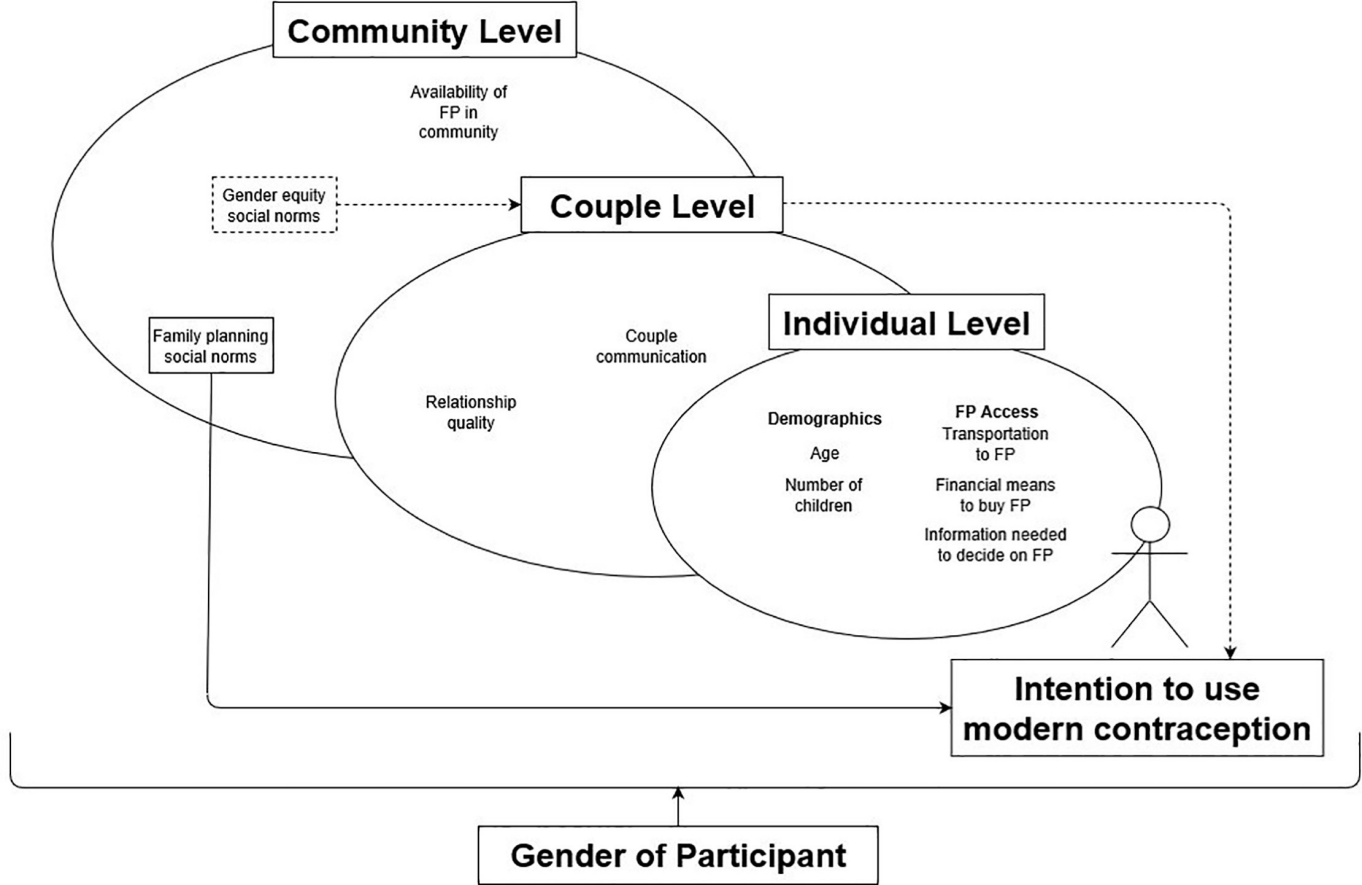
Conceptual model of factors theorized to affect use of modern contraception in Kinshasa, DRC.

As shown in Fig 1, we used a socioecological framework for this analysis with three levels of factors (individual, couple, and community) to conceptualize our expectations of which factors were likely to affect a respondent’s use of a modern contraception, as well as how these factors were likely to interact. At the individual level and based on previous research findings, we included in our model age, number of children, access to informational and financial resources, and access to FP services [23–25]. We expected that gender would have effects not only on the outcome but on all other variables (levels of influence) in this model, as demonstrated by placing gender in its own box at the bottom of the model. Although we theorized that the same set of factors would contribute to contraceptive use intentions among men and women, we expected that some of those factors might differ in strength, direction, or both according to gender. Previous literature has shown that men and women’s control over and interest in FP often differs among couples [26, 27] and is driven by different considerations [27–29]. At the couple level, previous research has shown that couples’ relationship quality (i.e., listening to their partner’s concerns during the past month) [30], as well as their communication and shared decision making (i.e., partners discussing their desired number of children) [25, 30], is associated with use of modern contraceptives.

Our interest was in measuring the extent to which community-level social norms existing prior to the MFF intervention were associated with intention to use modern contraceptives after controlling for select individual- and couple-level characteristics. In particular, we drew upon the conceptualization of Chung and Rimal [31] to devise questions to get at both descriptive and injunctive norms. Chung and Rimal portray descriptive norms as perceptions about whether others perform the behavior and injunctive norms as pressure to conform to norms either to avoid negative social consequences such as exclusion or sanctions or alternately to gain social approval [31, 32]. Consistent with the conceptualization of the intervention, we were interested in both the influence of proximal social norms (i.e., those related directly to FP use) and distal social norms (i.e., those indirectly related to FP use, in this case, those related to gender equity). In other words, in terms of the latter, we expected that individuals who reported believing in the existence of social norms around a more gender-equitable household (i.e., social approval of men’s involvement in child care and men’s involvement in household chores) would be more likely to experience improved communication with their spouse (i.e., couple communication). In turn, we anticipated that improved couple communication would likely affect the couple’s ability to discuss FP and may ultimately have a bearing on their intentions to use FP (i.e., indirect effects). While we expected both types of social norms to influence intentions to use modern contraceptives, we expected the proximal FP social norms to have a stronger influence on intentions than the more distal social norms about gender equity, as has been theorized by Cislaghi and Heise [33].

Establishing a baseline of the prevalence of these social norms and their influence on intention to use modern contraceptives prior to the intervention will better enable us to assess changes over time in this particular normative environment as a result of the intervention. In addition, we simultaneously lay the groundwork for the development of validated measures for social norms. The findings from this analysis will provide important insights for MFF programming and similar interventions focused on norms change and will contribute to a larger research agenda for improving the measurement and design of social-norms programming.

## Methods

### Data collection

The MFF intervention study utilizes a prospective, experimental, two group, pre-/post-test design. The Kinshasa School of Public Health and Georgetown University’s IRBs both reviewed and approved the protocol for this study. We conducted the baseline survey between September 2016 and February 2017 with 900 community members belonging to 17 communities serviced by MFF in Kinshasa; eight of these were subsequently randomized to immediately receive the intervention and nine to receive it after completion of the endline survey in late 2018. The intervention consists of a series of trainings and workshops that guide faith leaders and congregation members through a process of participatory reflections to identify, create, disseminate, embrace and take action on new, positive masculinities and gender equality.

The research team in cooperation with the churches developed sampling frame lists of all newly-married couple parishioners in participating churches in these 17 communities and subsequently data collectors attended church services on multiple occasions in an attempt to enroll at least one member of each eligible couple. Women were eligible if they were 18 to 35 years of age, reported that they had been in a relationship (either cohabitating and/or engaged) for no more than three years, and who had children less than three years of age. Men were eligible to participate if their female partner was age 18 to 35 and they were 18 or older. All who volunteered, gave their written consent, and met the eligibility criteria were accepted although data collectors monitored through their sampling frame lists that only one member of each couple participated in the survey. Data collectors were matched to the participant’s gender and administered the surveys on tablets in private locations near the church using a computer-assisted personal interviewing (CAPI) platform. Participants were offered refreshments but no monetary incentives.

### Survey instrument and measures

The survey (see S1 File and S2 File) included questions on demographics, attitudes, behaviors, and social norms. The survey was informed by two formative phases of research. One was undertaken in the Ituri Province in northeastern DRC prior to the original conceptualization of the Transforming Masculinities intervention approach, and the other was an exploration of social norms conducted in Kinshasa in early 2016. Both formative phases utilized participatory qualitative techniques and confirmed a range of social norms influential for target behaviors.

#### Individual- and couple-level factors

We also asked questions about the specific individual- and couple-level factors described in our conceptual model; these included individual-level demographic characteristics (e.g., age, number of children, and gender) and access to FP and couple-level characteristics such as relationship quality and communication. We assessed relationship quality with a set of four items related to relationship satisfaction. We assessed couple communication with a set of three items related to the couple’s recent discussions related to FP and fertility. All items on relationship quality and couple communication were asked with binary (yes/no) response options. Age and number of children were continuous variables, while all of the other individual- and couple-level factors were asked with binary (yes/no) response options.

#### Social norms

Based on findings from the formative phase of research and with an eye toward developing the evidence base for normative change [16, 34], the survey included an array of questions to elicit several types of social norms related to behaviors of interest. We asked questions both about perceptions of social approval (i.e., injunctive norms) and community prevalence (i.e., descriptive norms) relating to FP use and questions about social approval (i.e., injunctive norms) for engaging in tasks and roles traditionally ascribed to women—household chores and childcare responsibilities. Across the social norms variables, we inquired about the influence of a number of reference groups (i.e., different types of people thought to influence the social norm), including faith leaders, partners, and other NMCs and FTPs in the congregation. All social norms items were asked on four-point ordinal response scale (see S1 Table).

#### Outcome measure

For our outcome measure, we chose to analyze self-reported intention to use modern contraceptives in the future instead of current use of a modern contraceptive method. On the survey, respondents were asked how likely they were to use modern contraceptives in the future. This question was asked on a four-point Likert scale, with one being extremely unlikely to use and four being extremely likely to use modern contraceptives in the future. We modelled this question as a linear outcome. We decided to focus on future intention to use a modern contraceptive method for several reasons. First, because the intervention was focused on NMCs and FTPs, we expected that many of these couples would currently be trying to conceive, and we wanted to focus on motivating them to use FP to help space and time their future child bearing. In addition, we were assessing intention to use prior to initiation of the MFF intervention, recognizing that normative change takes time to achieve. We focused on modern contraceptive methods, as opposed to use of any contraceptive method, because the MFF intervention is particularly focused on promoting modern contraceptives over traditional or other forms of FP.

### Analysis plan

We developed a statistical analysis plan to identify the latent social norm constructs (variables that were not directly observed but rather were inferred from measured variables) by assessing which social norm survey items captured similar aspects, allowing us to determine their association with intention to use a modern contraceptive. To assist both with data reduction and to assess the latent structure underlying the social norm items, we first conducted exploratory factor analyses (EFAs) in IBM SPSS Statistics version 21 using maximum likelihood extractions with oblique (Promax) rotations. We conducted the EFAs with the entire sample to determine the latent constructs and retained items with factor loadings of 0.4 or higher. We used both the eigenvalues and scree tests in our initial determination of the number of social norm factors to retain. We reverse-scored any items that loaded negatively onto a factor to ensure that all items loaded in the same (positive) direction. We conducted two separate EFAs for FP and household gender equity, respectively. We included all survey items that were intended to analyze social norms, separating those about FP and those about household gender equity. We also used Cronbach’s Coefficient Alpha statistics to examine the internal consistency of each derived factor. We named each factor in a manner to align intuitively with the factor’s score; that is, a higher score onto a factor indicates greater agreement with that factor.

Subsequently, we fit separate structural equation models (SEM) for men and women to test whether the data supported our theoretical model. We believed that a SEM would be the most appropriate way to simultaneously assess the latent structure underlying sets of observed variables and the associations between those latent constructs and other variables. Prior to running the SEMs, we examined the distribution of each latent construct to be included in the model and all the pair-wise correlations between the variables of interest, latent and observed. Finally, we fit a series of SEMs using the Lavaan Package in R [35] to simultaneously assess the combination of latent and observed variables and their associations with each other and the outcome of interest. We iteratively allowed for inter-item associations to improve model fit and ultimately selected the model that was both parsimonious and best-fit, using the Comparative Fit Index (CFI) and the Root Mean Square Error of Approximation (RMSEA).

## Results

### Descriptive statistics

The majority of female survey respondents were 18 to 29 years of age, and most did not yet have any children with their current partner (Table 1). In contrast, half of the male respondents were over age 29 and were more educated than the female respondents, with 88% having completed higher than a secondary education. Survey respondents were almost evenly split between those who were in a relationship (35%), were engaged (38%), or were married (28%).

**Table 1:** Demographic characteristics of male and female survey respondents.

	Female (n = 493)		Male (n = 407)		Total (n = 900)	
	n	%	n	%	n	%
Age Group						
18–24	196	39.8	95	23.3	291	32.3
25–29	178	36.1	100	24.6	278	30.9
>29	119	24.1	206	50.6	325	36.1
Missing	0	0	6	1.5	6	0.7
Ethnicity						
Bakongo	284	57.6	219	53.8	503	55.9
Other	207	42.0	185	45.5	392	43.6
Missing	2	0.4	3	0.7	5	0.6
Urban vs. Peri-Urban						
Urban	278	56.4	247	60.7	525	58.3
Peri-urban	215	43.6	160	39.3	375	41.7
Education Status						
Less than secondary	80	16.2	9	2.2	89	9.9
Completed secondary	157	31.8	40	9.8	197	21.9
Higher than secondary	256	51.9	358	88.0	614	68.2
Problems Satisfying Food Needs in Past Year						
Never	269	54.6	175	43.0	444	49.3
Less than daily	141	28.6	154	37.8	295	32.8
Daily	77	15.6	75	18.4	152	16.9
Missing	6	1.2	3	0.7	9	1.0
Attend Church Every Week	386	78.3	323	79.4	709	78.8
Relationship Status						
In a relationship	166	33.7	147	36.1	313	34.8
Engaged	190	38.5	149	36.6	339	37.7
Married	137	27.8	111	27.3	248	27.6
Number of Children with Current Partner						
0	335	68.0	281	69.0	616	68.4
1	82	16.6	59	14.5	141	15.7
More than 1	66	13.4	53	13.0	119	13.2
Missing	10	2.0	14	3.4	24	2.7

In terms of FP use (Table 2), a little less than half of both male and female respondents at baseline reported that either they or their partner had ever used modern FP. Only about one-fifth reported that they were extremely likely to use a modern method in the future. In terms of access to FP, roughly three-quarters or more of participants said that modern methods were locally available to them and that they had the financial resources to purchase them, and over 70% reported having transportation to a facility where FP methods are available. In contrast, only about half reported that they had enough information to make a decision about which method to use. Respondents indicated that a variety of different types of reference groups influenced their FP use. While partners and spouses were the most commonly reported reference group (36%), mothers and mothers-in-law were the second most commonly reported reference group for FP decision making (28%), followed closely by faith leaders (28%) for both male and female respondents. Frequencies of reported reference groups were for the most part consistent across genders.

**Table 2:** Self-reported family planning use, intentions, self-efficacy, access, and reference groups among male and female survey respondents.

	Female (n = 493)			Male (n = 407)			Total (n = 900)	
	n	%		n	%		n	%
Ever use of modern contraception	241	48.9		187	45.9		428	47.6
Current use of any family planning method	262	53.1		219	53.8		481	53.4
Current use of modern contraceptive method^a^	168	34.1		141	34.6		309	34.3
Condom only	109	22.1		100	24.6		209	23.2
Other modern method	42	8.5		23	5.7		65	7.2
Likelihood of future use of modern contraception								
Extremely likely	94	19.1		99	24.6		193	21.4
Likely	271	55.1		209	51.9		480	53.3
Unlikely	65	13.2		46	11.4		111	12.3
Extremely unlikely	26	5.3		12	3.0		38	4.2
Missing	36	7.3		37	9.2		73	8.1
Family planning access								
Have modern methods available in this community	416	84.4		349	85.7		765	85.0
Have means to purchase modern methods	404	81.9		337	82.8		741	82.3
Have transportation/access to a provider	369	74.8		301	74.0		670	74.4
Have information needed for FP decision making	278	56.4		228	56.0		506	56.2
People whose opinions related to family planning matter to the respondent^b^								
Partner/spouse	202	41.0		124	30.5		326	36.2
Mother/mother-in-Law	154	31.2		98	24.1		252	28.0
Faith leader	124	25.2		125	30.7		249	27.7
Male relative	105	21.3		118	29.0		223	24.8
Friends	92	18.7		81	19.9		173	19.2
Medical professional	65	13.2		71	17.4		136	15.1
Other female relative	75	15.2		28	6.9		103	11.4
Other^c^	37	7.5		49	12.0		86	9.6

^a^ Modern contraceptive method is defined here as sterilization (male or female), contraceptive pills, IUDs, injectables, implants, diaphragms/foams/gels, Standard Days Method (SDM), lactational amenorrhea method (LAM), or condoms.

^b^ Participants were presented all options and allowed to choose as many as applied. All options were determined during the formative phase of the research.

^c^ Other includes godparents, members of the congregation, and other couples.

Regarding couple communication (Table 3), over 80% of both men and women had ever discussed the number of children they wanted to have with their partner. However, only about half had discussed the modern contraceptive method they would like to use with their partner, and less than half had discussed how to obtain modern contraception with their partner in the past year (Table 3). Reported relationship quality in the sample was high, with over three-quarters of respondents reporting that in the last month they had told their partner that they appreciated him or her, had taken time to listen to their partner’s concerns and talked with their partner about what made them happy. Only about two-thirds, however, had talked with their partner about what frustrates them.

**Table 3:** Couple communication and relationship quality among male and female survey respondents.

	Female (n = 493)		Male (n = 407)		Total (n = 900)	
	n	%	n	%	n	%
Couple communication						
Ever discussed the number of children you want to have with partner [q22]	403	81.7	329	80.8	732	81.3
Discussed with partner the modern FP method you would like to use in past year [q23]	227	46.0	210	51.6	437	48.6
Discussed with partner how to obtain modern FP method in past year [q24]	218	44.2	184	45.2	402	44.7
Relationship quality						
Told partner you appreciated them in past month [q18]	412	83.6	366	89.9	778	86.4
Took time to listen to partner’s concerns in past month [q19]	428	86.8	369	90.7	797	88.6
Talked about what makes you happy with partner in past month [q20]	437	88.6	362	88.9	799	88.8
Talked about what frustrates you with partner in past month [q21]	320	64.9	285	70.0	605	67.2

### Exploratory factor analyses

Results of the factor analysis revealed four distinct social norms constructs (Table 4): two related directly to FP use and two related to household gender equity. Seven social norms items that asked about injunctive FP norms (i.e., perceived approval of FP use) loaded onto the first construct, and two items designed to discern descriptive norms of FP use (i.e., perceptions of the prevalence of FP use in the congregation among NMCs and FTPs) loaded onto the second FP social norm construct. The FP injunctive norm construct included items about the respondent’s perceived approval of modern FP from the congregation, faith leaders, and their partner, in addition to whether important influencers’ opinions influenced the respondent’s personal view on modern FP.

**Table 4:** Factor loadings and communalities based on exploratory factor analysis for items related to family planning social norms among men and women.

	Family Planning Norms Factor Solution (n = 900)		
	Faith Community & Reference Group Approval of FP Use	Perceptions of Prevalence of FP Use in Congregation	Communality
	(α = 0.84)	(α = 0.83)	
Members of this congregation think it is appropriate for NMCs to use modern methods of FP. [q1]	0.58		0.32
Members of this congregation think it is appropriate for FTPs to use modern methods of FP. [q2]	0.64		0.46
Faith leaders in this congregation think it is appropriate for FTPs to use a modern method of FP. [q3]	0.66		0.48
Faith leaders think it is appropriate for NMCs to use a modern method of FP. [q4]	0.64		0.38
In matters related to FP, people whose opinions are important to me think I should use a modern method of FP. [q5]	0.67		0.44
My partner thinks we, as a couple, should use a modern method of FP. [q6]	0.70		0.47
Faith leaders in this congregation think my partner and I should use a modern method of FP. [q7]	0.70		0.48
Perceived proportion of congregation in which NMCs use a modern method of FP [q8]		0.69	0.52
Perceived proportion of congregation in which FTPs use a modern method of FP [q9]		†	1.00

†Contains Heywood case. Heywood cases occur when the item’s communality is greater than 1.0, which is an impossible outcome.

Similarly, the social norms related to gender equity loaded onto two constructs (Table 5), with the distinction in this case related to behaviors rather than type of norm (i.e., descriptive vs. injunctive). Four items assessing perceptions of social approval of men doing household chores loaded onto the first construct, and four items assessing perceptions of social approval of childcare responsibilities loaded onto the second construct. Notably, in this case, both constructs were comprised of items asking about injunctive norms and the reference groups referred to in these two sets were the same: faith leaders, partners, NMCs and FTPs in the congregation, and influential people.

**Table 5:** Factor loadings and communalities based on exploratory factor analysis for items related to social norms for gender equity among men and women.

	Household Gender Role Norms Factor Solution (n = 900)		
	Role in Chores	Role in Child Care	Communality
	(α = 0.86)	(α = 0.79)	
Most NMCs and FTPs that I know in this congregation approve of the husband sharing in the household work. [q10]	0.83		0.66
People whose opinions are important to me approve of the husband sharing in the household work. [q11]	0.78		0.58
Faith leaders in this congregation think my partner and I should both share in the house work. [q12]	0.77		0.65
My partner thinks we should both share in the housework. [q13]	0.70		0.55
My partner thinks we should both share in the responsibility of child care. [q14]		0.82	0.62
Faith leaders in this congregation think my partner and I should both share in the responsibility of child care. [q15]		0.73	0.55
Most NMCs and FTPs that I know in this congregation approve of the husband sharing in the responsibilities of child care. [q16]		0.62	0.47
People whose opinions are important to me, approve of the husband sharing in the responsibilities of child care. [q17]		0.52	0.35

For each of the four social norm constructs, all the underlying manifest variables had high factor loadings (i.e., all over 0.50), and all communalities were at or above 0.30. The Cronbach’s alphas for all factors were over 0.75, indicating the factors were internally consistent. (For more descriptive statistics of factors, see S2 Table).

### Structural equation models

**Fig 2:**
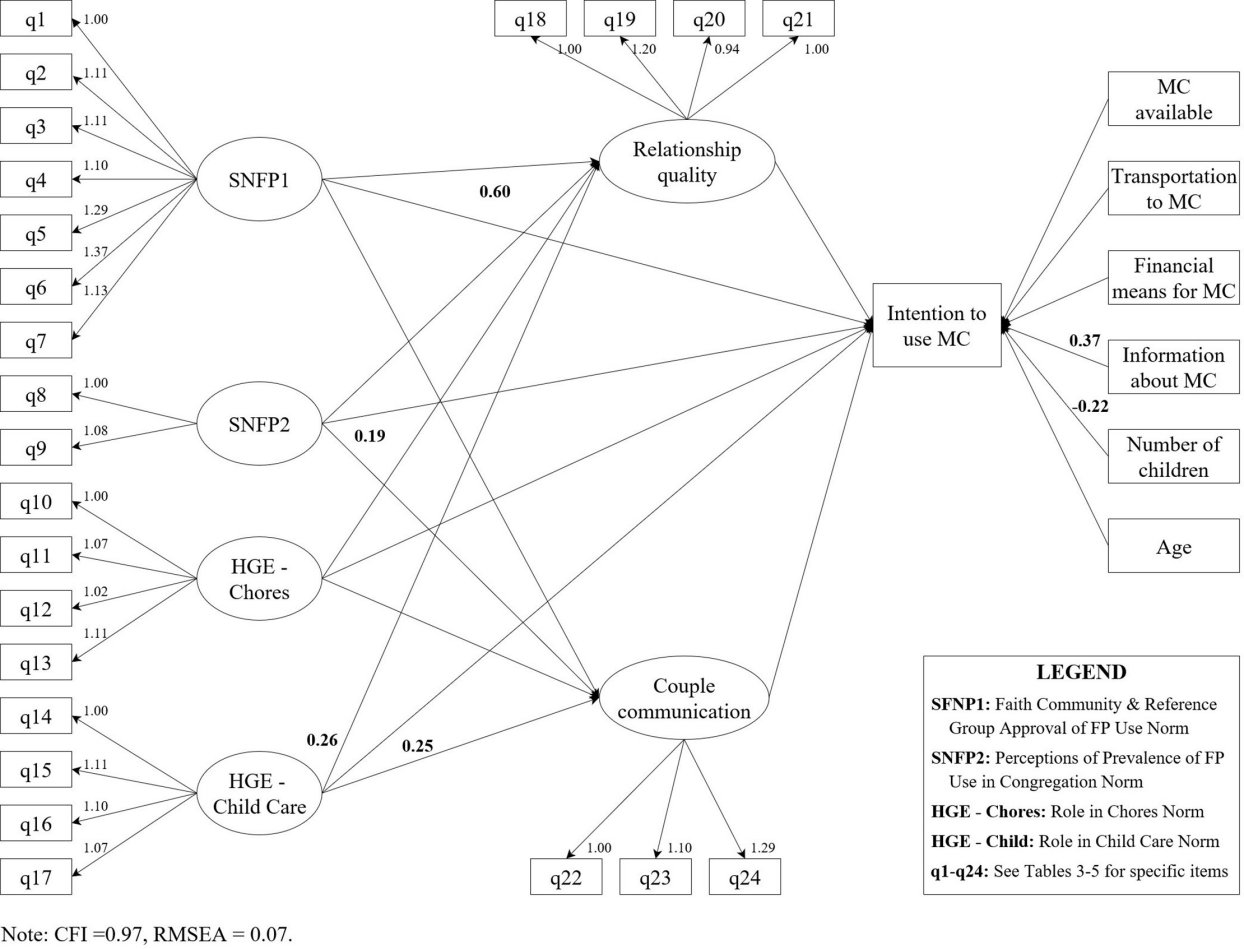
Structural equation model for the association of social norms with intention to use a modern contraceptive method among women, Kinshasa, DRC 2017.

**Fig 3:**
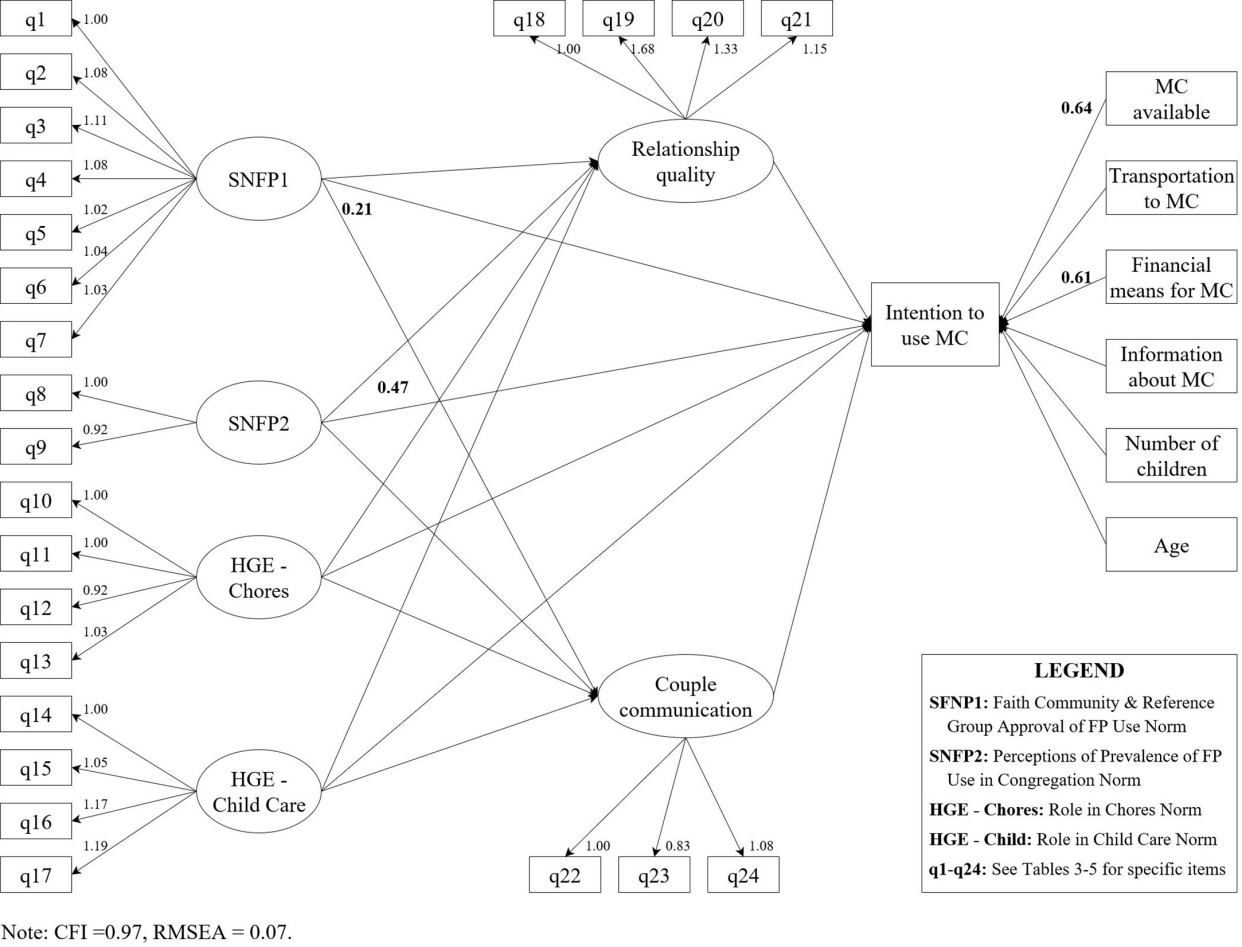
Structural equation model for the association of social norms with intention to use a modern contraceptive method among men, Kinshasa, DRC 2017.

Our SEMs for both genders fit the data well, as suggested by the CFI of 0.97 for both the women and the men and RMSEA of 0.07 for both the women and men (Figs 2 and 3). Furthermore, in both models all of the manifest variables loaded strongly onto their latent constructs), with all factor loadings being close to 1. We had hypothesized that the latent constructs would have a simple structure, and this was confirmed by the fact that each variable loaded onto only one factor.

Across the two models, we found that more than half of our hypothesized covariates had a significant association with our outcome of interest, but no one factor was statistically significant at the p < 0.05 level in both models. Therefore, in both the men’s and women’s models multiple factors were associated with intention to use family planning, there was not one common factor that was significantly associated with the outcome for both men and women. Common to both models was the fact that respondent age, having transportation to a FP clinic, relationship quality, and couple communication were not significantly associated with intention to use a modern method. In addition, none of the social norm constructs had significant indirect effects (i.e., via relationship quality or couple communication) on intention to use a modern method.

Among the women, the variables that were significantly associated with intention to use a modern contraceptive method were the number of children the respondent had, the information she had about FP, and perceptions of two social norms; injunctive FP norms and descriptive gender equity norms related to childcare responsibilities (Fig 2). All but the former were positively associated with intention to use a modern FP method. That is, we saw an increase in intention to use a modern method of about 0.4 for every one-unit increase in FP information (coefficient = 0.37; 95% CI: 0.03, 0.35). We also saw an increase in intention of 0.6 for every one-unit increase in injunctive FP norms (coefficient = 0.60; 95% CI: 0.42, 0.77), and an increase in intention of about 0.3 for every one-unit increase in injunctive gender equity norms (coefficient = 0.25; 95% CI: 0.01, 0.50). This can be interpreted as: for every one unit greater the belief that the faith community or reference group approve of FP use (i.e., injunctive FP norms), there was an increase of 0.6 in intention to use a modern method, and for every one unit greater the belief that people in the community approve of gender-equitable roles in child care (i.e., injunctive gender equity norms around child care), there was an increase of 0.3 in intention to use a modern method. Number of children, however, was negatively associated with FP intentions such that with every additional child a female respondent had, there was an accompanying approximate 0.2 (coefficient = -0.22; 95% CI: -0.37, -0.07) unit decrease in intention to use a modern method. In addition, we saw that descriptive FP norms were significantly associated with couple communication (coefficient = 0.19; 95% CI: 0.03, 0.35) among women; however, despite this positive effect, couple communication did not, in turn, have a significant association with intention to use a modern method.

Among the men, having the means to purchase contraception, an understanding of the availability of FP methods in the community, and perceptions of descriptive FP social norms were significantly associated with intention to use a modern method of contraception (Fig 3). Specifically, for every one-unit increase in means to purchase contraception, understanding of availability, and perceptions of descriptive FP norms, we saw an increase in intention to use a modern method of about 0.6 (coefficient 0.61; 95% CI: 0.18, 1.03), 0.6 (coefficient 0.64; 95% CI: 0.19, 1.09), and 0.5 (coefficient 0.47; 95% CI: 0.12, 0.83), respectively. This can be interpreted as for every one unit greater the belief that more people in the community were using FP (i.e., descriptive FP norms), there was an increase of 0.6 in intention to use a modern method. In addition, injunctive FP norms were also shown to be significantly positively associated with couple communication (coefficient = 0.21; 95% CI: 0.04, 0.38) among the men. However, in this population, neither couple communication nor relationship quality were significantly associated with intention to use a modern method. Notably, we found that for both the men and the women, the proximal FP social norms (i.e., norms related directly to community approval and use of FP) had stronger associations with our outcome than did the distal social norms on household gender equity, which was as we had hypothesized. (For more information on direct and indirect via mediation effect estimates of social norm constructs on intention to use family planning, see S3 Table).

## Discussion

While our findings are of greatest and most immediate relevance to the MFF intervention and similar normative change and FP interventions in the DRC, they also provide important insights for the broader community of scientists and applied practitioners seeking to measure and change social norms. In this analysis, we found that prior to any normative change activities, social norms were associated with intention to use modern contraception among young adults in this community in Kinshasa and that the social norms of relevance to FP intentions differed between men and women. Furthermore, we found that faith leaders and congregation members were important reference groups for FP matters in their respective communities. These findings suggest that the MFF intervention is conceptualized appropriately around the idea that changing social norms will in turn lead to normative environments more supportive of intentions to use modern contraceptive methods, as well as in its recognition and engagement of faith communities as instrumental to normative change for the study population.

This analysis is among the first to examine and show distinctions between the social norms most salient to men and to women. Specifically, we found that the descriptive norm around FP use was only influential for men, and that the injunctive norm related to FP use was only influential among the women. These results indicate that men are more swayed by their perceptions about how many of their peers are using FP, whereas women are more concerned about social sanctions resulting from use of FP. The social norm around gender equity in household chores was also only significant among women and not men, indicating that for women a key factor in increasing their interest in and use of modern FP methods lies in cultivating normative environments supportive of men partaking equally in domestic responsibilities.

A critical question in programs targeting normative change is who are the most important reference groups that should be included and targeted with norms-change programming. Our findings provide some insight, albeit limited, into this question. The FP descriptive norm that we identified was the only social norm construct that was specific to a distinct reference group—in this case, other congregation members who were NMCs and FTPs. This finding is useful in its indication that in terms of behavioral role models, congregation members in this context who are NMCs and FTPs look to others in their life stage and religious community rather than looking more broadly at older generations or individuals outside their religious community. We also found some evidence that faith leaders were an important reference group in this population (i.e., faith leaders were reported by many as an influential group and loaded onto three of the four social norms constructs). The other three social norms constructs included multiple reference groups, however. For instance, the injunctive norms constructs relating to gender equity in household chores and childcare responsibilities included items that referred to NMCs and FTPs in their congregation, faith leaders, their partner, and influential influencers. The mixture of multiple reference groups in one construct makes findings more complicated to interpret and translate into practice; this is because it is not clear if all of the reference groups have an equal influence on creation of the social norm or practice of the behavior and because it adds additional components and target groups to the intervention, rendering it more complex, costly, and difficult to scale up.

We are aware of a number of limitations to our survey and analysis. First, on the multiple-choice survey question asking respondents to identify whose opinions related to FP mattered to them (Table 2), 23% of respondents specified a relation other than the response options provided, and of these, 65% wrote in some type of medical professional (i.e., doctor, nurse, etc.). Close to 30% of respondents also indicated that a mother or mother-in-law was a person whose opinions related to FP mattered to them. Unfortunately, neither medical professionals nor mothers and mothers-in-law were included as influential reference groups in the central intervention activities or in the questions in the social norms survey for MFF. Given their importance, greater incorporation of these reference groups into the central intervention activities could serve to augment the impact of the intervention, inasmuch as theory suggests that the motivation to comply with a social norm is greater if the norms come from people important to an individual [31, 36, 37].

In addition, a few of our findings were contrary to our expectations, and not all of the factors that we hypothesized would be associated with intentions to use were, in fact, significant. Availability of FP methods and having the means to purchase contraception were important factors for men and not for women. Previous research has shown that parity is a significant influence on couples (and in particular, women) to both desire to use and actually use FP, with women who have a greater number of children being more likely to desire and use FP [38–40]. In contrast, we found that in this population, the number of children female respondents had was inversely related to intention to use modern contraception. We think that this is likely an artifact of the focus in this intervention on NMCs and FTPs. Very few participants had more than one child (13%), such that those who had children were, therefore, likely to be at the very beginning stages of starting their family and currently actively trying to have children. Individuals at other life stages, such as older adults, younger adults and adolescents, were not included in the survey or targeted for the intervention, thus eliminating the possibility of comparing the influence of these factors across different life stages. Related, and for similar reasons, age was not significant in either of our models (but this was likely due to the fact that that the survey targeted a largely similar age range of respondents).

Also unexpected was the fact that social norms did not exert significant indirect effects on intention to use via influencing relationship quality and couple communication. In fact, in this population, neither relationship quality nor couple communication had a significant influence on our outcome of interest. We suspect that this is likely due to the fact that close to 80% of participants reported engaging in behaviors indicative of good relationship quality and couple communication at baseline. These high reported levels of couple communication and good relationship quality may in turn be related to the fact that this sample was more educated on average than the general population [41]; a factor that has also been shown to have a positive correlation with more gender equitable attitudes [11]. Notably however less than half of women (i.e., 41%) and only a third of men (31%) named their partner/spouse as a person whose opinion related to family planning matters to them. This is consistent with the literature previously cited on gender equitable beliefs in the DRC and may also contribute to why relationship quality and couple communication were not significant in our models.

Also of note, whereas social norms related to men participating in childcare responsibilities were significant in the women’s model, perceptions of social approval of men sharing equally in domestic responsibilities were not significant in either model (a significant influence on FP intentions for either the men or the women). These findings could indicate that FP behaviors in this population are largely sustained by more proximal social norms or that the questions related to household gender equity social norms were not the distal norms that exerted the greatest influence on FP behaviors in this population. It could also be that survey respondents were aware of the intervention’s goals and, as such, provided more socially desirable responses leading to an overestimation of both intention to use and actual use of modern contraception in Kinshasa’s population (e.g., 30% [our population] vs. 19% [Kinshasa]) [6].

Our findings indicate not only that social norms are influential in both men and women’s FP behavior, but also that it is necessary to disentangle which norms are most salient to each gender. Indeed, the fact that different social norms came into play among the men and the women has important implications for MFF and similar interventions focusing on changing social norms that affect use of FP methods. In the current design of the MFF intervention, some of the activities are conducted separately with men and women, but additional differentiation of messaging and incorporation of key reference groups by participant gender may be a modification worth considering for future iterations of this intervention. Doing so will also help FP programs foster normative environments that are supportive of FP use for both men and women, as well as facilitate more fine-tuned indicators for future social norms measurement.

Currently, preparations are being made to scale up the MFF intervention beginning with the nine control sites and then expanding to 30 additional congregations in Kinshasa Province by 2020. Moreover in light of the fact that religious leaders throughout the developing world, regardless of denomination, are influential community figures, we feel optimistic that the MFF intervention could be successfully adapted and scaled up to a variety of other religious communities and community settings throughout the developing world.

The government of the DRC has committed to achieving a 19% modern contraceptive prevalence rate and to expanding access to FP services to at least 2.1 million additional women of reproductive age by 2020 [8]. To reach these ambitious goals, FP programs in the DRC will need to go beyond supply-side issues. In the DRC context of heightened GBV and widespread religiosity, more positive social and gender norms need to be promulgated to facilitate improved social acceptance of contraceptive use; churches and faith leaders will be critical allies in this endeavor. This analysis contributes important evidence to support the need for interventions targeting changes in social norms, as well as to more appropriately target messages related to social norms to men and to women.

## Supporting information

S1 File Transforming masculinities baseline survey–women’s survey. (PDF)

S2 File Transforming masculinities baseline survey–men’s survey. (PDF)

S1 Table Descriptive statistics of items included in exploratory factor analysis. (DOCX)

S2 Table Descriptive statistics of latent constructs in structural equation models. (DOCX)

S3 Table Total effects of social norm constructs on intention to use modern family planning, separated by direct effects of social norms on outcome and indirect effects via mediation of couple’s relationship quality and communication. (DOCX)
