# Supplementary material for: Getting to intent: Are social norms influencing intentions to use modern contraception in the DRC?
Source: PLoS One. 2019 Jul 16;14(7):e0219617. doi: 10.1371/journal.pone.0219617 (PMC6634398; doi:10.1371/journal.pone.0219617)
Supplement: S1 File — (PDF) [file pone.0219617.s001.pdf]

Transforming Masculinities
Baseline Survey
Women's Survey

Today's date _______ / _______ / _______
Day Month Year

Section 1: Background Characteristics. Let's start with some questions about you:

No. (Tablet)	Questions and filters	Coding categories	Skip to
**SECTION 1: Background characteristics**			
(1)	Consent form		
(2)	Interviewer code		

No. (Tablet)	Questions and filters	Coding categories	Skip to
(3)	What is the name of the church where you are a member?	Pretest<br>Paroisse de la Communauté des Eglises en Mission (CEM/NEST)……………………………………1<br><br>Aumônerie Universitaire Protestante de Kinshasa (AUPK) paroisse de l'Université Pédagogique de Kinshasa (UPN)……………..2<br><br>Paroisse Internationale Protestante de Kinshasa…………………………………………3<br><br>Aumônerie Universitaire Protestante de Kinshasa (AUPK), Université de Kinshasa (Unikin), Paroisse Satellite de Matete………..4<br><br>Paroisse de l'Eglise du Rocher de la Communauté Evangélique de l'Alliance au Congo (CEAC)…………………………………..5<br><br>Paroisse de Kimvula de la Communauté Baptiste Congo Ouest (CBCO)………………..6<br><br>Paroisse de Lemba-Salongo, Communauté Presbytérienne de Kinshasa…………………7<br><br>Paroisse de Masina, Communauté Evangélique du Kwango (CEK)……………..8<br><br>Paroisse N'Djili de la Communauté Evangélique au Congo (CEC)……………….9<br><br>Paroisse Bumbu 1 de la Communauté Baptiste du Congo Ouest (CBCO)……………………10<br><br>Paroisse de Makala 3 de la Communauté Baptiste du Congo Ouest (CBCO)…………11<br><br>Paroisse de la Chapelle de la Victoire de la Communauté des Assemblées de Dieu en Afrique (CADAF)………………………………12<br><br>Paroisse de Mont - Ngafula de la Communauté des Eglises Libres de Pentecôte en Afrique (CELPA)………………………………………13<br><br>Paroisse de l'ozone de la Communauté Evangélique de l'Alliance au Congo (CEAC)…………………………………………14<br><br>Paroisse de Lisala de la Communauté Baptiste du Fleuve Congo (CBFC)……………………15<br><br>Paroisse de Bandalungwa de la Communauté Des Eglises de Pentecôte en Afrique Centrale (CEPAC)……………………………………16<br><br>Paroisse Saint Pierre de l'Eglise Anglicane du Congo (EAC)…………………………………17	

No. (Tablet)	Questions and filters	Coding categories	Skip to
(4)	Thank you again for agreeing to speak with me today. Let's start with a few questions about you		
(5)	Has your partner participated in this survey in the last 14 days?	Yes……………………………………1<br>No……………………………………..2<br>Don't know…………………………..99	
101 (6)	How old are you? *(If she does not know her age*: "*Can you tell me in what year were you born*?" AGE TO BE CALCULATED AFTER INTERVIEW.)	Age .... ☐☐<br>Year born.......................... ☐☐☐☐	
102 (7)	What is the highest level of school you have attended?	Never attended school .... 1<br>Incomplete primary..................................... 2<br>Completed primary .... 3<br>Incomplete secondary .... 4<br>Completed secondary .... 5<br>Higher than secondary ………………………6	
103 (8)	What is your relationship status?	In a relationship, but not engaged or married…………………………………………..1<br>Engaged to be married…………………..……2<br>Married…………………………………………3	
104 (9)	Are you and your partner living together as a couple?	Yes………………………………………1<br>No……………………………………..2	
105 (10)	How old were you when you started living together with your partner as a couple?	Years: _____ ☐☐<br>If less than one year, record the number of months: _______ ☐☐	
106 (11)	How many children have you given birth to who are alive?	Number of living children.................. ☐☐	If the response is "0", go to Q108
107 (12)	How many of these children are with your current partner?	Number of living children………….. ☐☐	

No. (Tablet)	Questions and filters	Coding categories	Skip to
108 (13)	What is your religion?	Catholic...1<br>Protestant ....2<br>Kimbanguiste …………………………………..3<br>Other Christian …………………………………4<br>Muslim …………………………………………..5<br>Orthodox...6<br><br>Other …………………………..……………..88<br>(specify)____________________	
109 (14)	How would you describe your typical attendance at your church for services or prayers?	Attend services or prayers every week…….…1<br>Attend services or prayers every few months………………………………………….…..2<br>Attend services or prayers on special days only (Easter, Christmas, etc)…………………….3<br>Never attend services or prayers………..…….4<br>No response……………………………….…..77<br>Other…………………………………………..88<br>Specify______________________________	
110 (15)	How important is your religion to you?	Very important ………………………………1<br>Important……………………………………..2<br>Not very important…………………………3<br><br>No response………………………………77	
111 (16)	What is your ethnicity?	Luba ……………………………….…………… 1<br>Mongo…………………………………………....2<br>Bakongo ………………………………………….3<br>Mangbetu-Azande……………………………..4<br>Other ………………………………..……….88	

No. (Tablet)	Questions and filters	Coding categories	Skip to
112 (17)	Who are the adults that live in the same household with you and your partner?<br><br>MULTIPLE OPTIONS POSSIBLE.	Mother-in-law……………………………………1<br>Father-in-law……………………….……………2<br>Mother……………………………………………3<br>Father……………………………..……………..4<br>Uncle in-law………..……………..….…………5<br>Aunt in-law………………………………………6<br>Uncle ………………………………..………..…7<br>Aunt……………………………………………...8<br>Other male relative……………………………..9<br>Other female relative…………………..……..10<br><br>Other (specify)________________________88	
113 (18)	How often has your household had problems in satisfying the food needs in the last 12 months?<br><br>CHOOSE ONLY ONE OPTION	Never ……………………………………….…..1<br>Once or more in a year ………………………2<br>Once in a month ……………………….…..3<br>Once in a week ……………………..……….4<br>Daily ………………………………...………..5	
**SECTION 2: Family Planning**			
(19)	Now I would like to talk about family planning – the ways or methods that a couple can use to delay or avoid a pregnancy		
201 (20)	Are you pregnant, or do you think you might be pregnant?	Yes ....1<br>No ....2<br>Not sure 8	If Yes, go to →Q205
202 (21)	Are you currently doing something or using any method to delay or avoid getting pregnant?	Yes ....1<br>No 2	Go to →Q.204

No. (Tablet)	Questions and filters	Coding categories	Skip to
203<br>(22)	Which method are you using?<br><br>MULTIPLE RESPONSES POSSIBLE. DO NOT READ THE LIST. CIRCLE THE LETTER FOR EACH MENTIONED.	Female sterilization ....A<br>Male sterilization..B<br>Pill.. C<br>IUD .... D<br>Injectables ....E<br>Implants...F<br>Condom.. G<br>Diaphragm/foam/jelly.................................... H<br>Standard Days Method/CycleBeads ....I<br>Lactational Amenorrhea Method .... J<br>Periodic abstinence/calendar method ....K<br>Withdrawal...L<br>Herbal preparations (drink)............................M<br>Herbal preparations (douche)........................ N<br>Spider web.. O<br>Beads/amulets...P<br>Other……………………………………………X<br>_______________________<br>(specify)	
204<br>(23)	You have said you are **not** using any method to avoid pregnancy.<br><br>Could you tell me why you are not using a method?<br><br>Any other reason?<br><br>MULTIPLE RESPONSES POSSIBLE. DO NOT READ THE LIST. CIRCLE THE LETTER FOR EACH MENTIONED.	**FERTILITY-RELATED REASONS**<br>I want to have a child………………………..A<br>Infrequent/not having sex ....B<br>I can't get pregnant .... C<br>I have not menstruated since last birth ..... D<br>I am breastfeeding ....E<br>Up to God/fatalistic ....F<br><br>**OPPOSITION TO USE**<br>Respondent opposed .... G<br>Husband opposed .... H<br>Others opposed ....I<br>Religious prohibition .... J<br>Encourage promiscuity………………………K<br><br>**LACK OF KNOWLEDGE**<br>Knows no method ....L<br>Knows no source ....M<br><br>**METHOD-RELATED REASONS**<br>Side effects/health concerns .... N<br>Methods are not 100% effective……………O<br>Health concerns (child) ....P<br>Lack of access/too far .... Q<br>Costs too much .... R<br>Preferred method not available ....S<br>No method available ....T<br>Inconvenient to use .... U<br>Other…………………………………………..X<br>_______________________<br>(specify)<br>Do not know..Z	

No. (Tablet)	Questions and filters	Coding categories	Skip to
205<br>(24)	Have you ever done or used any method to delay or avoid getting pregnant?	Yes .... 1<br>No 2	 Go to Q.207
206<br>(25)	Which method have you used in the past?<br><br>CIRCLE ALL MENTIONED	Female sterilization .... A<br>Male sterilization .... B<br>Pill .... C<br>IUD .... D<br>Injectables .... E<br>Implants .... F<br>Condom .... G<br>Diaphragm/foam/jelly .... H<br>Standard Days Method/CycleBeads .... I<br>Lactational Amenorrhea Method .... J<br>Periodic abstinence/calendar method .... K<br>Withdrawal .... L<br>Herbal preparations (drink) .... M<br>Herbal preparations (douche) .... N<br>Spider web .... O<br>Beads/amulets .... P<br>Other .... X<br>____________________<br>(specify)	
207<br>(26)	How likely would you say it is that you will use a modern method of family planning in the future, extremely likely, likely, unlikely or extremely unlikely?	Extremely likely .... 1<br>Likely .... 2<br>Unlikely .... 3<br>Extremely unlikely .... 4<br>Not applicable 77	
**Access to Family Planning**			
(27)	I'm now going to read you a series of statements regarding access to family planning. For each statement, I will ask you to respond yes or no.		
208<br>(28)	Modern methods of family planning are available in the community where I live.	Yes .... 1<br>No 2	
209<br>(29)	I have transportation to get to a health clinic, pharmacy, or Community Health Worker that provides family planning methods.	Yes .... 1<br>No 2	
210<br>(30)	I have the means to purchase modern methods of family planning if I want to use them.	Yes .... 1<br>No 2	
211<br>(31)	I have enough information to make a decision about which modern method to use if I wanted to use one.	Yes .... 1<br>No 2	
212<br>(32)	My husband will give me money to purchase a modern method of family planning.	Yes .... 1<br>No 2	
**SECTION 3: Family Planning Norms**			

No. (Tablet)	Questions and filters	Coding categories	Skip to
**Family Planning: Outcome Expectations**			
(33)	I will read several statements. For each statement please let me know how much you agree with the statement – strongly agree, agree, disagree or strongly disagree.		
301 (34)	If I use a modern method of family planning (for example condoms, Jadelle, sterilization, etc.) I will avoid an unwanted pregnancy.	Strongly agree ……………………..................1<br>Agree…………………….…………….……….2<br>Disagree.……………………………………..….3<br>Strongly disagree ………………………………4	
302 (35)	If I use a modern method of family planning I will have difficulty becoming pregnant in the future.	Strongly agree ……………………..................1<br>Agree…………………….…………….……….2<br>Disagree.……………………………………..….3<br>Strongly disagree ………………………………4	
303 (36)	If I use a modern method of family planning it would not be against my religion.	Strongly agree ……………………..................1<br>Agree…………………….…………….……….2<br>Disagree.……………………………………..….3<br>Strongly disagree ………………………………4	
304 (37)	If I use a condom I will have less sexual pleasure.	Strongly agree ……………………..................1<br>Agree…………………….…………….……….2<br>Disagree.……………………………………..….3<br>Strongly disagree ………………………………4	
305 (38)	If I use a modern method of family planning I will get a reputation for being promiscuous.	Strongly agree ……………………..................1<br>Agree…………………….…………….……….2<br>Disagree.……………………………………..….3<br>Strongly disagree ………………………………4	
306 (39)	If I use a modern method of family planning I will experience negative side effects.	Strongly agree ……………………..................1<br>Agree…………………….…………….……….2<br>Disagree.……………………………………..….3<br>Strongly disagree ………………………………4	
307 (40)	If I mention using a modern method of family planning with my husband, he will have a negative reaction.	Strongly agree ……………………..................1<br>Agree…………………….…………….……….2<br>Disagree.……………………………………..….3<br>Strongly disagree ………………………………4	
**Family Planning: Attitudes, Injunctive Norms, and Intentions**			

No. (Tablet)	Questions and filters	Coding categories	Skip to
(41)	I will read several statements. For each statement, consider your own feelings about these matters and tell me what you think. I will read each statement and the response options.		
308<br>(42)	For me, avoiding an unwanted pregnancy is…	Extremely good……….………...……………..1<br>Good...………….………..…..………………….2<br>Bad…...…………………….………………….3<br>Extremely bad…………………..………………4	
309<br>(43)	For me, sexual pleasure is…	Extremely important……..……….……………..1<br>Important…………….……………….……….2<br>Unimportant…..…………………………….…….3<br>Extremely unimportant………………………….4	
310<br>(44)	For my husband (partner), to use a modern method of family planning is…	Extremely valuable to hi health……………..…1<br>Valuable to his health………………………….2<br>Not very valuable to his health………………..3<br>Not at all valuable to his health………………..4	
311<br>(45)	For me, to use a modern method of family planning is ….	Extremely valuable to my own health……….…..…1<br>Valuable to my own health………………………….2<br>Not very valuable to my own health………………..3<br>Not at all valuable to my own health………………..4	
312<br>(46)	For me, experiencing negative side effects of a modern method of family planning is …	Extremely likely…………………….................1<br>Likely…………………………..………………..2<br>Unlikely.………..……………………………….3<br>Extremely unlikely…………………………….4	
**Family Planning: Self-efficacy**			
(47)	Now I will read a few statements. For each one, I want you to tell me how confident you feel you could do each of these things.		
313a<br>(48)	I can use a modern method of family planning correctly to avoid or delay a pregnancy.	Very confident…………………………..……..1<br>Confident…………………………………………2<br>Not very confident……………………………….3<br>Not at all confident…………………………….4	

No. (Tablet)	Questions and filters	Coding categories	Skip to
313b (49)	I can use a modern method of family planning correctly all the time to delay or avoid pregnancy, even if the faith leaders in my church disagree	Very confident……………………………….1<br>Confident……………………………………2<br>Not very confident………………………………3<br>Not at all confident………………………………4	
313c (50)	I can use a modern method of family planning correctly all the time to delay or avoid pregnancy.	Very confident……………………………….1<br>Confident……………………………………2<br>Not very confident………………………………3<br>Not at all confident………………………………4	
313d(51)	I can suggest using a modern method of family planning to my partner.	Very confident……………………………….1<br>Confident……………………………………2<br>Not very confident………………………………3<br>Not at all confident………………………………4	
**Family Planning: Injunctive Norms**			
(52)	For this next set of questions, I will read a series of statements. For each statement, I want to know what you think people expect others to do. You can say strongly agree, agree, disagree or strongly disagree.		
314 (53)	Members of this congregation think it is appropriate for newly married couples to use modern methods of family planning	Strongly agree ………………………..................1<br>Agree……………………………………………..2<br>Disagree.…………………………………………3<br>Strongly disagree ………………………………4	
315 (54)	Members of this congregation think it is appropriate for first time parents to use modern methods of family planning	Strongly agree ………………………..................1<br>Agree……………………………………………..2<br>Disagree.…………………………………………3<br>Strongly disagree ………………………………4	
316 (55)	Faith leaders in this congregation think it is appropriate for first time parents to use a modern method of family planning	Strongly agree ………………………..................1<br>Agree……………………………………………..2<br>Disagree.…………………………………………3<br>Strongly disagree ………………………………4	
317 (56)	Faith leaders think it is appropriate for newly married couples to use a modern method of family planning	Strongly agree ………………………..................1<br>Agree……………………………………………..2<br>Disagree.…………………………………………3<br>Strongly disagree ………………………………4	
318 (57)	It is appropriate for newly married couples to use a modern method of family planning.	Strongly agree ………………………..................1<br>Agree……………………………………………..2<br>Disagree.…………………………………………3<br>Strongly disagree ………………………………4	

No. (Tablet)	Questions and filters	Coding categories	Skip to
319 (58)	It is appropriate for first time parents to use a modern method of family planning.	Strongly agree ……………………..................1<br>Agree……………………….…………………..2<br>Disagree…………………………………..….3<br>Strongly disagree ………………………………4	
**Family Planning: Normative Beliefs (Subjective Norms)**			
320 (59)	In matters related to family planning, whose opinion matters is important to you?<br><br>DO NOT READ OPTIONS<br>CHECK ALL OPTIONS THAT APPLY	Husband……………………………………… 1<br>Friends…………………………………………2<br>Mother…………………………………………3<br>Father…………………………………………..4<br>Mother-in-law…………………………………..5<br>Father-in-law…………………………………..6<br>Faith leader……………………………………7<br>Sister……………………………………………8<br>Brother…………………………………………9<br>Other female relative…………………………10<br>Other male relative……………………………11<br>Other……………………………………………88<br>Specify______________________________	
321 (60)	In matters related to family planning, people whose opinions are important to me think I should use a modern method of family planning.	Strongly agree ……………………..................1<br>Agree……………………….…………………..2<br>Disagree………………………………………3<br>Strongly disagree ………………………………4	
322 (61)	My husband (partner) thinks we, as a couple, should use a modern method of family planning.	Strongly agree ……………………..................1<br>Agree……………………….…………………..2<br>Disagree…………………………………..….3<br>Strongly disagree ………………………………4	
323 (62)	Faith leaders in this congregation think my husband (partner) and I should use a modern method of family planning.	Strongly agree ……………………..................1<br>Agree……………………….…………………..2<br>Disagree…………………………………..….3<br>Strongly disagree ………………………………4	
**Family Planning: Motivation to Comply**			
324 (63)	In matters related to family planning, how important is it for you to do what your husband wants you to do?	Very important…………………………………1<br>Important………………………………………..2<br>Not so important…...…………………………..3<br>Not at all important……………………………4	

No. (Tablet)	Questions and filters	Coding categories	Skip to
325 (64)	In matters related to family planning, how important is it for you to do what the faith leaders in this congregation want you to do?	Very important……………………………………1<br>Important………………………………………..2<br>Not so important…...…………………………..3<br>Not at all important……………………………4	
326 (65)	In matters related to family planning, how important is it for you to do what people whose opinion matter to you want you to do?	Very important……………………………………1<br>Important………………………………………..2<br>Not so important…...…………………………..3<br>Not at all important……………………………4	
**Family Planning: Descriptive Norms**			
(66)	*Now I would like to know what people in your congregation actually do. Think about what is normal or typical behavior when you respond to these statements. Do you think that these statements are true for most, many, some or none of the people in this congregation?*		
327 (67)	Newly married couples in your congregation use a modern method of family planning.	None ……………………………...................1<br>Some ……………..…………………….……….2<br>Many ……………..……………………….…..….3<br>Most ……………………..………………..……..4	
328 (68)	First time parents in your congregation use a modern method of family planning.	None ……………………………...................1<br>Some ……………..…………………….……….2<br>Many ……………..……………………….…..….3<br>Most ……………………..………………..……..4	
**SECTION 4: Couple Communication and Family Planning Decision-Making**			
(69)	I'm now going to read you a series of statements regarding communicating with your partner and making family planning decisions. You can respond yes or no.		
401 (70)	Have you ever discussed with your husband (partner) the number of children you would like to have?	Yes……………………………………….……….1<br>No ……………………………………..…….…….2	
402 (71)	In the last 12 months, have you discussed with your husband (partner) the type of family planning method you would like to use to avoid or delay pregnancy?	Yes……………………………………….……….1<br>No ……………………………………..…….…….2	
403 (72)	In the last 12 months, have you discussed with your husband (partner) how to obtain a family planning method to avoid or delay pregnancy?	Yes……………………………………….……….1<br>No ……………………………………..…….…….2	
404 (73)	If you and your husband disagree about using a modern method of family planning, who makes the final decision?	Me……………………………………….…….....1<br>My husband……………………….……………..2<br>Both decide together ………………..……...3	
**SECTION 5: Intimate Partner Violence**			

No. (Tablet)	Questions and filters	Coding categories	Skip to
(74)	*The next questions are about things that happen to many women and men. To answer these questions, we will use some cards. Each card contains a word that can describe some actions that you might have experienced. I also have three envelopes here. They say 'never', 'sometimes', and 'often'. Please pick up each card and look at the action. Put the card in the envelope that best describes the frequency of that action. I am turning my back away so that I cannot see where you are placing your card.*		
501	Has your husband/partner done the following things to you in the last 12 months…		
501a (75)	Shouted or yelled at you?	Often…………………………………...…………1<br>Sometimes ……………………………………2<br>Never ……………………………………….………3<br>No response ………………………..………..77	
501b (76)	Threatened to hurt you or someone you care about?	Often…………………………………...…………1<br>Sometimes ……………………………………2<br>Never ……………………………………….………3<br>No response ………………………..………..77	
501c (77)	Pushed you, shaken you, or thrown something at you?	Often…………………………………...…………1<br>Sometimes ……………………………………2<br>Never ……………………………………….………3<br>No response ………………………..………..77	
501d (78)	Slapped you or twisted your arm?	Often…………………………………...…………1<br>Sometimes ……………………………………2<br>Never ……………………………………….………3<br>No response ………………………..………..77	
501e (79)	Punched you with his fist or with something else that could hurt you?	Often…………………………………...…………1<br>Sometimes ……………………………………2<br>Never ……………………………………….………3<br>No response ………………………..………..77	
	Within the last 12 months …..		
502a (80)	Has your partner physically forced you to have sexual intercourse when you did not want to?	Often…………………………………...…………1<br>Sometimes ……………………………………2<br>Never ……………………………………….………3<br>No response ………………………..………..77	
502b (81)	Has your partner yelled or used physical force to discourage you from using a modern method of family planning?	Often…………………………………...…………1<br>Sometimes ……………………………………2<br>Never ……………………………………….………3<br>No response ………………………..………..77	
**Justification of Intimate Partner Violence**			
(82)	*Sometimes a husband is annoyed or angered by things that his wife does. In your opinion is a husband justified in hitting or beating his wife if she…*		
503	In your opinion is a husband justified in hitting or beating his wife if she…		
503a (83)	Goes out without telling him?	Yes ………………………………………….………1<br>No …………………………………………...…..2<br>No response ……………………………..…..77	

No. (Tablet)	Questions and filters	Coding categories	Skip to
503b (84)	Neglects the children?	Yes ……………………………………….………1<br>No ……………………………………………..….2<br>No response ……………………………..…..77	
503c (85)	Argues with him?	Yes ……………………………………….………1<br>No ……………………………………………..….2<br>No response ……………………………..…..77	
503d (86)	Refuses to have sex with him?	Yes ……………………………………….………1<br>No ……………………………………………..….2<br>No response ……………………………..…..77	
503e (87)	Burns the food?	Yes ……………………………………….………1<br>No ……………………………………………..….2<br>No response ……………………………..…..77	
503f (88)	Uses a modern method of family planning without his knowledge	Yes ……………………………………….………1<br>No ……………………………………………..….2<br>No response ……………………………..…..77	
(89)	Now I want to ask you a few questions about when you were a child. Think about the time before you were 15 years old and living with parents or another caretaker.		
504 (90)	Did you see or hear your mother or other women in your home being beaten by your father or another man living in the home?	Never……………………………………………1<br>Sometimes………………………………………2<br>Often……………………………………………..3	
505 (91)	Were you threatened with physical punishment in your home?	Never……………………………………………1<br>Sometimes………………………………………2<br>Often……………………………………………..3	
506 (92)	Did your parents or caretakers or any other adults in the home slap or beat you?	Never……………………………………………1<br>Sometimes………………………………………2<br>Often……………………………………………..3	
**SECTION 6: Intimate Partner Violence Norms**			
	**Intimate Partner Violence: Outcome Expectations**		
(93)	*I'm now going to read you a series of statements regarding violence by a husband against his wife, and then I will ask you if you strongly agree, agree, disagree or strongly disagree on each of them.*		
601 (94)	A husband beats his wife to correct her bad behavior.	Strongly agree……………………………………1<br>Agree....……………………….…………………2<br>Disagree……………………………………….3<br>Strongly disagree………………………………4	
602 (95)	A husband beating his wife is a normal part of married life.	Strongly agree……………………………………1<br>Agree....……………………….…………………2<br>Disagree……………………………………….3<br>Strongly disagree………………………………4	

No. (Tablet)	Questions and filters	Coding categories	Skip to
603 (96)	If the neighbors see or hear a husband beating his wife, they will try to stop him	Strongly agree……………………………………1<br>Agree....……………………….……………….2<br>Disagree…………………………………………..3<br>Strongly disagree…………………………………4	
**Intimate Partner Violence: Attitudes, Perceived Control, and Intentions**			
(97)	*I want to read to you a few more statements. Please tell me how much you agree with each statement. You can say strongly agree, agree, disagree or strongly disagree.*		
604 (98)	According to the scripture a husband is supposed to discipline his wife.	Strongly agree……………………………………1<br>Agree....……………………….……………….2<br>Disagree…………………………………………..3<br>Strongly disagree…………………………………4	
605 (99)	For my husband to give equal weight to what I say in making decisions is….to me.	Extremely important…………………………..….1<br>Important……………….………………………2<br>Unimportant……………………………………....3<br>Extremely unimportant………………………..…4	
606 (100)	If a man does not beat his wife, people will think he is not manly.	Strongly agree……………………………………1<br>Agree....……………………….……………….2<br>Disagree…………………………………………..3<br>Strongly disagree…………………………………4	
607 (101)	I would use other nonviolent strategies to manage conflict with my husband (partner) if I knew them.<br><br>*Please tell me how likely it is for you to do this.*	Strongly agree……………………………………1<br>Agree....……………………….……………….2<br>Disagree…………………………………………..3<br>Strongly disagree…………………………………4	
**Intimate Partner Violence: Injunctive Norms**			
(102)	For this next set of questions, I will read a series of statements. For each statement, I want to know what you think people expect others to do. You can say strongly agree, agree, disagree or strongly disagree.		
608 (103)	People in this congregation expect a husband to force his wife to have sex even when she does not want to	Strongly agree…………………………………..1<br>Agree……………………………………………2<br>Disagree…………………………………………3<br>Strongly disagree………………………………4	
609 (104)	People in this congregation think it is ok for a husband to beat his wife at times	Strongly agree…………………………………..1<br>Agree……………………………………………2<br>Disagree…………………………………………3<br>Strongly disagree………………………………4	

No. (Tablet)	Questions and filters	Coding categories	Skip to
610 (105)	Faith leaders think it is ok for a husband to beat his wife at times	Strongly agree……………………………………..1<br>Agree…………………………………………….2<br>Disagree…………………………………………3<br>Strongly disagree………………………………4	
611 (106)	Faith leaders think it is ok for a husband to force his wife to have sex even when she does not want to	Strongly agree……………………………………..1<br>Agree…………………………………………….2<br>Disagree…………………………………………3<br>Strongly disagree………………………………4	
612 (107)	It is appropriate for a husband to beat his wife at times	Strongly agree……………………………………..1<br>Agree…………………………………………….2<br>Disagree…………………………………………3<br>Strongly disagree………………………………4	
**Intimate Partner Violence: Subjective Norms**			
613 (108)	For matters related to my relationship with my husband, whose opinion matters to you?	Husband……………………………………… 1<br>Friends…………………………………………2<br>Mother……………………………………………3<br>Father…………………………………………..4<br>Mother-in-law…………………………………..5<br>Father-in-law…………………………………..6<br>Faith leader……………………………………7<br>Sister……………………………………………8<br>Brother…………………………………………9<br>Other female relative…………………………10<br>Other male relative……………………………11<br>Other……………………………………………88<br>Specify________________________	
614 (109)	My husband thinks it is ok for him to beat me at times.	Strongly agree……………………………………1<br>Agree....…………………….………………..2<br>Disagree……………………………………..3<br>Strongly disagree………………………………4	
615 (110)	My husband thinks it is ok for him to force me to have sex even when I do not want to.	Strongly agree……………………………………1<br>Agree....…………………….………………..2<br>Disagree……………………………………..3<br>Strongly disagree………………………………4	

No. (Tablet)	Questions and filters	Coding categories	Skip to
616 (111)	Faith leaders in this congregation think it is ok for my husband to beat me at times.	Strongly agree……………………………………1<br>Agree….…………………….………………….2<br>Disagree…………………………………………..3<br>Strongly disagree……………………………….4	
617 (112)	People whose opinion is important to me think it is ok for my husband to beat me at times.	Strongly agree……………………………………1<br>Agree….…………………….………………….2<br>Disagree…………………………………………..3<br>Strongly disagree……………………………….4	
**Intimate Partner Violence: Motivation to Comply**			
618 ((113)	In matters of my relationship with my husband, I want to do what my husband wants me to do.	Strongly agree……………………………………1<br>Agree….…………………….………………….2<br>Disagree…………………………………………..3<br>Strongly disagree……………………………….4	
619 (114)	In matters of my relationship with my husband, I want to do what faith leaders in this congregation want me to do.	Strongly agree……………………………………1<br>Agree….…………………….………………….2<br>Disagree…………………………………………..3<br>Strongly disagree……………………………….4	
**Intimate Partner Violence: Descriptive Norms**			
(115)	*Now I would like to know what people in your congregation actually do. Think about what is normal or typical behavior when you respond to these statements. Do you think that these statements are true for most, many, some or none of the newly married couples or first time parents in this congregation?*		
620 (116)	A husband beats his wife	None…….…………………………………………1<br>Some…………………………..……………………2<br>Many……………………..……………………….3<br>Most……………………………………………….4	
621 (117)	A husband forces his wife to have sex even when she does not want it	None…….…………………………………………1<br>Some…………………………..……………………2<br>Many……………………..……………………….3<br>Most……………………………………………….4	
**SECTION 7: Gender Roles and Positive Masculinities**			
**Gender and Positive Masculinities: Attitudes, Injunctive Norms and Intentions**			

No. (Tablet)	Questions and filters	Coding categories	Skip to
(118)	*I want to read to you a few more statements. Please tell me whether you how much you agree with the statement.*		
701 (119)	My husband gives equal weight to what I say when making a decision	Strongly agree……………………………………1<br>Agree.....…………………….……………………2<br>Disagree…………………………………………..3<br>Strongly disagree…………………………………4	
702 (120)	People whose opinions are important to me, approve of the husband sharing in the household work such as doing dishes, cleaning and cooking.	Strongly agree……………………………………1<br>Agree.....…………………….……………………2<br>Disagree…………………………………………..3<br>Strongly disagree…………………………………4	
703 (121)	I believe men and women were created equal.	Strongly agree……………………………………1<br>Agree.....…………………….……………………2<br>Disagree…………………………………………..3<br>Strongly disagree…………………………………4	
704 (122)	I can express my opinion even if my husband disagrees.	Strongly agree……………………………………1<br>Agree.....…………………….……………………2<br>Disagree…………………………………………..3<br>Strongly disagree…………………………………4	
705 (123)	People whose opinions are important to me, approve of husbands sharing in the responsibilities of child care	Strongly agree……………………………………1<br>Agree.....…………………….……………………2<br>Disagree…………………………………………..3<br>Strongly disagree…………………………………4	
706 (124)	It is important to me that my husband plays a role in bringing up our children beyond being the financial provider.	Strongly agree……………………………………1<br>Agree.....…………………….……………………2<br>Disagree…………………………………………..3<br>Strongly disagree…………………………………4	
707 (125)	Most newly married couples and first time parents that I know in this congregation approve of the husband sharing in the household work such as washing dishes, cleaning and cooking	Strongly agree……………………………………1<br>Agree.....…………………….……………………2<br>Disagree…………………………………………..3<br>Strongly disagree…………………………………4	
708 (126)	Most newly married couples and first time parents that I know in this congregation approve of the husband sharing in the responsibilities of child care.	Strongly agree……………………………………1<br>Agree.....…………………….……………………2<br>Disagree…………………………………………..3<br>Strongly disagree…………………………………4	
**Normative Beliefs (Subjective Norms)**			

No. (Tablet)	Questions and filters	Coding categories	Skip to
709 (127)	My husband thinks we should both share in the house work such as doing dishes, cleaning and cooking.	Strongly agree……………………………………1<br>Agree.....……………………….……………….2<br>Disagree…………………………………………..3<br>Strongly disagree……………………………….4	
710 (128)	My husband thinks we should both share in the responsibility of child care.	Strongly agree……………………………………1<br>Agree.....……………………….……………….2<br>Disagree…………………………………………..3<br>Strongly disagree……………………………….4	
711 (129)	Faith leaders in this congregation think my husband and I should both share in the responsibility of child care.	Strongly agree……………………………………1<br>Agree.....……………………….……………….2<br>Disagree…………………………………………..3<br>Strongly disagree……………………………….4	
712 (130)	Faith leaders in this congregation think my husband and I should both share in the house work such as doing dishes, cleaning and cooking.	Strongly agree……………………………………1<br>Agree.....……………………….……………….2<br>Disagree…………………………………………..3<br>Strongly disagree……………………………….4	
**Gender and Positive Masculinities: Descriptive Norms**			
(131)	*Now I would like to know what people in your congregation actually do. Think about what is normal or typical behavior when you respond to these statements. Do you think that these statements are true for most, many, some or none of the people in this congregation?*		
713 (132)	The husband shares in the work around the house such as doing dishes, cleaning and cooking.	None ……………………………...................1<br>Some ……………..…………………….……….2<br>Many ………………..………………….….….3<br>Most ……………………..…………………..……..4	
714 (133)	The husband shares in the responsibilities of child care.	None ……………………………...................1<br>Some ……………..…………………….……….2<br>Many ………………..………………….….….3<br>Most ……………………..…………………..……..4	
**SECTION 9: Relationship Quality**			
(134)	*Now I want to ask you a few questions about your relationship with your husband (partner).*		
801 (135)	In the last month, did you tell your husband that you appreciated him?	Yes……………………………………….……….1<br>No ……………………………………………..2	
802 (136)	In the past month, did you take time to listen to your husband's concerns?	Yes…………………………………………..1<br>No ……………………………………………..2	

No. (Tablet)	Questions and filters	Coding categories	Skip to
803 (137)	In the past month, did you and your husband talk about things that frustrated you?	Yes……………………………………………1<br>No …………………………………….……….2	
804 (138)	In the past month, did you and your husband talk about things that made you happy?	Yes………………………………………………..1<br>No ………………………………………………..2	

Thank you for participating in this study!
