# Supplementary material for: Getting to intent: Are social norms influencing intentions to use modern contraception in the DRC?
Source: PLoS One. 2019 Jul 16;14(7):e0219617. doi: 10.1371/journal.pone.0219617 (PMC6634398; doi:10.1371/journal.pone.0219617)
Supplement: S2 File — (PDF) [file pone.0219617.s002.pdf]

Transforming Masculinities
Baseline
Men's Survey

Today's date _______ / _______ / _______
Day Month Year

Section 1: Background Characteristics. Let's start with some questions about you:

No. (Tablet)	Questions and filters	Coding categories	Skip to
**SECTION 1: Background characteristics**			
(1)	Consent form	Yes……………………………………………1<br>No…………………………………………….2	
(2)	Interviewer code	Interviewer code: ____________________	

No. (Tablet)	Questions and filters	Coding categories	Skip to
(3)	What is the name of the church where you are a member?	Pretest<br>Paroisse de la Communauté des Eglises en Mission (CEM/NEST)……………………………………1<br><br>Aumônerie Universitaire Protestante de Kinshasa (AUPK) paroisse de l'Université Pédagogique de Kinshasa (UPN)……………..2<br><br>Paroisse Internationale Protestante de Kinshasa……………………………………………3<br><br>Aumônerie Universitaire Protestante de Kinshasa (AUPK), Université de Kinshasa (Unikin), Paroisse Satellite de Matete………..4<br><br>Paroisse de l'Eglise du Rocher de la Communauté Evangélique de l'Alliance au Congo (CEAC)…………………………………..5<br><br>Paroisse de Kimvula de la Communauté Baptiste Congo Ouest (CBCO)………………..6<br><br>Paroisse de Lemba-Salongo, Communauté Presbytérienne de Kinshasa…………………7<br><br>Paroisse de Masina, Communauté Evangélique du Kwango (CEK)……………..8<br><br>Paroisse N'Djili de la Communauté Evangélique au Congo (CEC)……………….9<br><br>Paroisse Bumbu 1 de la Communauté Baptiste du Congo Ouest (CBCO)……………………10<br><br>Paroisse de Makala 3 de la Communauté Baptiste du Congo Ouest (CBCO)…………11<br><br>Paroisse de la Chapelle de la Victoire de la Communauté des Assemblées de Dieu en Afrique (CADAF)………………………………12<br><br>Paroisse de Mont - Ngafula de la Communauté des Eglises Libres de Pentecôte en Afrique (CELPA)………………………………………13<br><br>Paroisse de l'ozone de la Communauté Evangélique de l'Alliance au Congo (CEAC)………………………………………14<br><br>Paroisse de Lisala de la Communauté Baptiste du Fleuve Congo (CBFC)……………………15<br><br>Paroisse de Bandalungwa de la Communauté Des Eglises de Pentecôte en Afrique Centrale (CEPAC)………………………………………16<br><br>Paroisse Saint Pierre de l'Eglise Anglicane du Congo (EAC)…………………………………17	

No. (Tablet)	Questions and filters	Coding categories	Skip to
(4)	Thank you again for agreeing to speak with me today. Let's start with a few questions about you		
(5)	Has your partner participated in this survey in the last 14 days?	Yes…………………………………….1<br>No……………………………………..2<br>Don't know…………………………..99	
101 (6)	How old are you? *(If he does not know his age*: "*Can you tell me in what year were you born*?" AGE TO BE CALCULATED AFTER INTERVIEW.)	Age .... ☐☐<br>Year born......................... ☐☐☐☐	
102 (7)	What is the highest level of school you have attended?	Never attended school .... 1<br>Incomplete primary...................................... 2<br>Completed primary...................................... 3<br>Incomplete secondary .... 4<br>Completed secondary .... 5<br>Higher than secondary ………………………6	
103 (8)	What is your relationship status?	In a relationship, but not engaged or married…………………………………………1<br>Engaged to be married………………………..2<br>Married…………………………………………..3	
104 (9)	Are you and your partner living together as a couple?	Yes…………………………………………….1<br>No……………………………………………..2	
105 (10)	How old were you when you started living with your partner as a couple?	Years:___ ☐☐<br>If less than one year, record the number of months___________ ☐☐	
106 (11)	How many living children do you have?	Number of living children.................. ☐☐	If no children, put "0" and go to Q108
107 (12)	How many of these children have you had with your current partner?	Number of living children…………. ☐☐	

No. (Tablet)	Questions and filters	Coding categories	Skip to
108 (13)	What is your religion?	Catholic... 1<br>Protestant .... 2<br>Kimbanguiste ………………………………….3<br>Other Christian ……………………………….4<br>Muslim …………………………………………..5<br>Orthodox... 6<br>Other …………………………..……………..88<br>(specify)____________________	
109 (14)	How would you describe your typical attendance at your church for services or prayers?	Attend services or prayers every week……...1<br>Attend services or prayers every few months……………………………………….…..2<br>Attend services or prayers on special days only (Easter, Christmas, etc)………………..……..…3<br>Never attend services or prayers………..……4<br>No response……………………………….…..77<br>Other…………………………………………..88<br>Specify______________________________	
110 (15)	How important is your religion to you?	Very important ……………………………1<br>Important…………………………………..2<br>Not very important ………………………….3<br>No response…………………………….…77	
111 (16)	What is your ethnicity?	Luba ………………………………….…………… 1<br>Mongo………………………………………….....2<br>Bakongo …………………………………………3<br>Mangbetu-Azande…………………………….4<br>Other ……………………………………………..88	
112 (17)	Who are the adults that live in the same household with you/you and your partner?<br><br>MULTIPLE OPTIONS POSSIBLE.	Mother-in-law………………………………….1<br>Father-in-law…………………….…………….2<br>Mother…………………………………………..3<br>Father……………………………..…………….4<br>Uncle in-law……….………………..…………….5<br>Aunt in-law……………………………………….6<br>Uncle ………………………………..……….…7<br>Aunt……………………………………………...8<br>Other male relative……………………………..9<br>Other female relative…………………..……..10<br><br>Other (specify)_______________________88	

No. (Tablet)	Questions and filters	Coding categories	Skip to
113 (18)	How often has the household had problems in satisfying the food needs in the last 12 months?	Never ……………………………………..…….1<br>Once or more in a year ……………….……….2<br>Once in a month ……………………..……….3<br>Once in a week ……………………..……….4<br>Daily …………………………………………..5	
**SECTION 2: Family Planning**			
(19)	Now I would like to talk to you about family planning – the ways or methods couples use to delay or avoid pregnancy		
201 (20)	Is your wife pregnant, or do you think she is pregnant?	Yes .... 1<br>No .... 2<br>Not sure 8	If Yes, go to Q205
202 (21)	Are you or your wife currently doing something or using any method to delay or avoid getting pregnant?	Yes .... 1<br>No .... 2 →	Go to Q204
203 (22)	Which method are you or your wife using?<br><br>MULTIPLE RESPONSES POSSIBLE.<br>DO NOT READ THE LIST.<br>CIRCLE THE LETTER FOR EACH MENTIONED.	Female sterilization ....A<br>Male sterilization..B<br>Pill... C<br>IUD .... D<br>Injectables ....E<br>Implants ....F<br>Condom .... G<br>Diaphragm/foam/jelly.................................. H<br>Standard Days Method/CycleBeads .... I<br>Lactational Amenorrhea Method .... J<br>Periodic abstinence/calendar method ....K<br>Withdrawal... L<br>Herbal preparations (drink)........................... M<br>Herbal preparations (douche)....................... N<br>Spider web.. O<br>Beads/amulets..P<br>Other ……………………………………………X<br>____________________<br>(specify)	
204 (23)	You have said that you are not currently using a method to avoid pregnancy. Could you tell me why you are not using a method?<br><br>Any other reason?	**FERTILITY-RELATED REASONS**<br>I would like to have a child…………………A<br>Infrequent/not having sex ....B<br>Wife can't get pregnant .... C<br>Wife has not menstruated since last birth . D<br>Wife breastfeeding ....E<br>Want more children before using FP...........F<br>Up to God/fatalistic .... G	

No. (Tablet)	Questions and filters	Coding categories	Skip to
	MULTIPLE RESPONSES POSSIBLE.<br>DO **NOT** READ THE LIST.<br>CIRCLE THE LETTER FOR EACH MENTIONED.	**OPPOSITION TO USE**<br>Respondent opposed .... H<br>Wife opposed ....I<br>Others opposed .... J<br>Religious prohibition ....K<br>Encourage promiscuity..........................L<br><br>**LACK OF KNOWLEDGE**<br>Knows no method ....M<br>Knows no source .... N<br><br>**METHOD-RELATED REASONS**<br>Side effects/health concerns .... O<br>Methods are not 100% effective...............P<br>Health concerns (child) .... Q<br>Lack of access/too far .... R<br>Costs too much ....S<br>Preferred method not available ....T<br>No method available .... U<br>Inconvenient to use .... R<br>Other ………………………………………...X<br>________________________<br>(specify)<br><br>Do not know..Z	
205 (24)	Have you or your wife ever done or used any method to delay or avoid getting pregnant?	Yes .... 1<br>No 2	If No, Go to Q207
206 (25)	Which method have you used in the past?<br><br>CIRCLE ALL MENTIONED	Female sterilization ....A<br>Male sterilization...B<br>Pill.. C<br>IUD .... D<br>Injectables ....E<br>Implants ....F<br>Condom .... G<br>Diaphragm/foam/jelly.................................... H<br>Standard Days Method/CycleBeads ....I<br>Lactational Amenorrhea Method .... J<br>Periodic abstinence/ calendar method ....K<br>Withdrawal... L<br>Herbal preparations (drink)...........................M<br>Herbal preparations (douche)........................ N<br>Spider web.. O<br>Beads/amulets..P<br>Other …………………………………………X<br>________________________<br>(specify)	

No. (Tablet)	Questions and filters	Coding categories	Skip to
207 (26)	How likely would you say it is that you and your wife would use a modern method of family planning in the future?	Extremely likely……………………..................1<br>Likely…………………………….……………….2<br>Unlikely………..………………………………..3<br>Extremely unlikely………………………………4<br>Not applicable……………………………….77	
**Access to Family Planning**			
(27)	*I'm now going to read you a series of statements regarding access to family planning. For each statement, I will ask you to respond yes or no. When I say modern method of family planning, I mean a method that you have obtained in a pharmacy, health facility or from a Community Health Worker.*		
208 (28)	Modern methods of family planning are available in the community where I live.	Yes .... 1<br>No 2	
209 (29)	I have transportation to get to a health facility that provides family planning methods.	Yes .... 1<br>No 2	
210 (30)	I have the means to purchase modern methods of family planning if I want to use them.	Yes .... 1<br>No 2	
211 (31)	I have enough information to make a decision about which modern method to use if I wanted to use one.	Yes .... 1<br>No 2	
212 (32)	I will give my wife money to purchase a modern method of family planning.	Yes .... 1<br>No 2	
**SECTION 3: Family Planning Norms**			
**Family Planning Outcome Expectations**			
(33)	*I will read several statements. For each statement please let me know how much you agree with the statement – strongly agree, agree, disagree or strongly disagree.*		
301 (34)	If I use a modern method of family planning (for example a condom, Jadelle, sterilization, etc.) I will avoid an unwanted pregnancy.	Strongly agree ……………………..................1<br>Agree…………………………….……………….2<br>Disagree……………………………….………..3<br>Strongly disagree ………………………………4	
302 (35)	If I use a modern method of family planning I will have difficulty getting my wife pregnant in the future.	Strongly agree ……………………..................1<br>Agree…………………………….……………….2<br>Disagree……………………………….………..3<br>Strongly disagree ………………………………4	

No. (Tablet)	Questions and filters	Coding categories	Skip to
303 (36)	If I use a modern method of family planning, it would not be against my religion.	Strongly agree ……………………...............1<br>Agree…………………………….…………….2<br>Disagree………………………………….…….3<br>Strongly disagree ……………………………4	
304 (37)	If I use a condom I will have less sexual pleasure.	Strongly agree ……………………...............1<br>Agree…………………………….…………….2<br>Disagree………………………………….…….3<br>Strongly disagree ……………………………4	
305 (38)	If I use a modern method of family planning I will get a reputation for being promiscuous.	Strongly agree ……………………...............1<br>Agree…………………………….…………….2<br>Disagree………………………………….…….3<br>Strongly disagree ……………………………4	
306 (39)	If I use a modern method of family planning I will experience negative side effects.	Strongly agree ……………………...............1<br>Agree…………………………….…………….2<br>Disagree………………………………….…….3<br>Strongly disagree ……………………………4	
307 (40)	If my wife mentions using a modern method of family planning I will support her.	Strongly agree ……………………...............1<br>Agree…………………………….…………….2<br>Disagree………………………………….…….3<br>Strongly disagree ……………………………4	
**Family Planning: Attitudes, Injunctive Norms and Intentions**			
(41)	*I will read several statements. For each statement, consider your own feelings about these matters and tell me what you think. I will read each statement and the response options.*		
308 (42)	For me, avoiding an unwanted pregnancy is…	Extremely good……..………..……………….1<br>Good...…………..…………………………..…2<br>Bad….………………………………………….3<br>Extremely bad………………………………..4	
309 (43)	For me, sexual pleasure is…	Extremely important……..……………………..1<br>Important……………….………………….……..2<br>Unimportant……………………………….…….3<br>Extremely unimportant………………………….4	
310 (44)	For my wife (partner), to use a modern method of family planning is ….	Extremely valuable to my wife's health……….1<br>Valuable to my wife's health…………………..2<br>Not very valuable to my wife's health………..3<br>Not at all valuable to my wife's health………..4	

No. (Tablet)	Questions and filters	Coding categories	Skip to
311 (45)	For me, to use a modern method of family planning is ….	Extremely valuable to my own health……….1<br>Valuable to my own health……………….…….2<br>Not very valuable to my own health………..3<br>Not at all valuable to my own health………..4	
312 (46)	For my wife, experiencing negative side effects of a modern method of family planning is …	Extremely likely…………………...................1<br>Likely……………………….…………………..2<br>Unlikely………..……………………………….3<br>Extremely unlikely………………………………4	
**Family Planning Self efficacy**			
(47)	*Now I will read a few statements. For each one, I want you to tell me how confident you feel you could do each of these things.*		
313a (48)	I can use a modern method of family planning correctly to avoid or delay a pregnancy.	Very confident…………………………….………..1<br>Confident…………………………………………2<br>Not very confident………………………………3<br>Not at all confident………………………………4	
313b (49)	I can use a modern method of family planning correctly all the time to delay or avoid pregnancy, even if the faith leaders in my church disagree.	Very confident…………………………….………..1<br>Confident…………………………………………2<br>Not very confident………………………………3<br>Not at all confident………………………………4	
313c (50)	I can use a modern method of family planning correctly all the time to delay or avoid pregnancy.	Very confident…………………………….………..1<br>Confident…………………………………………2<br>Not very confident………………………………3<br>Not at all confident………………………………4	
313d (51)	I can suggest using a modern method of family planning to my partner.	Very confident…………………………….………..1<br>Confident…………………………………………2<br>Not very confident………………………………3<br>Not at all confident………………………………4	
**Family Planning: Injunctive Norms**			
(52)	*For this next set of questions, I will read a series of statements. For each statement, I want to know what you think people expect others to do. You can say strongly agree, agree, disagree or strongly disagree.*		
314 (53)	Members of this congregation think it is appropriate for newly married couples to use modern methods of family planning	Strongly agree ……………………..................1<br>Agree………………………….…………………..2<br>Disagree……………………………….………….3<br>Strongly disagree ………………………………4	

No. (Tablet)	Questions and filters	Coding categories	Skip to
315 (54)	Members of this congregation think it is appropriate for first time parents to use modern methods of family planning	Strongly agree ……………………………………1<br>Agree……………………………………………2<br>Disagree…………………………………………3<br>Strongly disagree ………………………………4	
316 (55)	Faith leaders in this congregation think it is appropriate for first time parents to use a modern method of family planning	Strongly agree ……………………………………1<br>Agree……………………………………………2<br>Disagree…………………………………………3<br>Strongly disagree ………………………………4	
317 (56)	Faith leaders think it is appropriate for newly married couples to use a modern method of family planning	Strongly agree ……………………………………1<br>Agree……………………………………………2<br>Disagree…………………………………………3<br>Strongly disagree ………………………………4	
318 (57)	It is appropriate for newly married couples to use a modern method of family planning.	Strongly agree ……………………………………1<br>Agree……………………………………………2<br>Disagree…………………………………………3<br>Strongly disagree ………………………………4	
319 (58)	It is appropriate for first time parents to use a modern method of family planning.	Strongly agree ……………………………………1<br>Agree……………………………………………2<br>Disagree…………………………………………3<br>Strongly disagree ………………………………4	
**Family Planning: Normative Beliefs**			
320 (59)	In matters related to family planning, whose opinion matters to you?<br><br>DO NOT READ OPTIONS.<br>CHECK ALL OPTIONS THAT APPLY.	Wife…………………………………… 1<br>Friends…………………………………………2<br>Mother…………………………………………3<br>Father…………………………………………4<br>Mother-in-law……………………………….5<br>Father-in-law………………………………..6<br>Faith leader…………………………………7<br>Sister……………………………………………8<br>Brother…………………………………………9<br>Other female relative………………………10<br>Other male relative…………………………11<br>Other……………………………………………88<br>Specify______________________________	

No. (Tablet)	Questions and filters	Coding categories	Skip to
321 (60)	In matters related to family planning, people whose opinions are important to me think my wife (partner) and I should use a modern method of family planning.	Strongly agree ……………………................1<br>Agree…………………………….………………2<br>Disagree………………………………………..3<br>Strongly disagree ……………………………4	
322 (61)	My wife (partner) thinks we, as a couple, should use a modern method of family planning.	Strongly agree ……………………................1<br>Agree…………………………….………………2<br>Disagree………………………………………..3<br>Strongly disagree ……………………………4	
323 (62)	Faith leaders in this congregation think my wife (partner) and I should use a modern method of family planning.	Strongly agree ……………………................1<br>Agree…………………………….………………2<br>Disagree.……………………………….….…..3<br>Strongly disagree ……………………………4	
**Family Planning: Motivation to Comply**			
324 (63)	In matters related to family planning, how important is it for you to do what your wife (partner) wants you to do?	Very important.……………………………….1<br>Important.……………………………………..2<br>Not so important.…..…………………………..3<br>Not at all important.…………………………..4	
325 (64)	In matters related to family planning, how important is it for you to do what the faith leaders in this congregation want you to do	Very important.……………………………….1<br>Important.……………………………………..2<br>Not so important.…..…………………………..3<br>Not at all important.…………………………..4	
326 (65)	In matters related to family planning, how important is it for you to do what people whose opinion matters want you to do?	Very important.……………………………….1<br>Important.……………………………………..2<br>Not so important.…..…………………………..3<br>Not at all important.…………………………..4	
**Descriptive Norms on Family Planning**			
(66)	*Now I would like to know what people in your congregation actually do. Think about what is normal or typical behavior when you respond to these statements. Do you think that these statements are true for most, many, some or none of the people in this congregation?*		
327 (67)	Newly married couples in your congregation use a modern method of family planning.	None ………………………….....................1<br>Some ……………..………………….……….2<br>Many ………………..……………….………..3<br>Most ………………….……………..……..4	

No. (Tablet)	Questions and filters	Coding categories	Skip to
328 (68)	First time parents in your congregation use a modern method of family planning.	None ……………………………………...................1<br>Some ……………..………………………..……….2<br>Many ………………..…………………….……….3<br>Most ……………………..……………….…..……..4	
**SECTION 4: Couple Communication and Family Planning Decision-Making**			
(69)	*I'm now going to read you a series of statements regarding communicating with your partner and making family planning decisions*		
401 (70)	Have you ever discussed with your wife (partner) the number of children you would like to have?	Yes………………………………………….……….1<br>No ……………………………….……..……..…….2	
402 (71)	In the last 12 months, have you discussed with your wife (partner) the type of family planning method you would like to use to avoid or delay pregnancy?	Yes………………………………………….……….1<br>No ……………………………….……..……..…….2	
403 (72)	In the last 12 months, have you discussed with your wife (partner) how to obtain a family planning method to avoid or delay pregnancy?	Yes………………………………………….……….1<br>No ……………………………….……..……..…….2	
404 (73)	If you and your wife disagree about using a modern method of family planning, who makes the final decision?	Me……………………………………….……….....1<br>My wife………………………….……………….2<br>Both decide together ……………………..……...3	

**SECTION 5: Intimate Partner Violence**			
(74)	*The next questions are about things that happen to many women and men. To answer these questions, we will use some cards. Each card contains a word that can describe some actions that you might have undertaken. I also have three envelopes here. They say 'never', 'sometimes', and 'often'. Please pick up each card and look at the action. Put the card in the envelope that best describes the frequency of that action. I am turning my back away so that I cannot see where you are placing your card).*<br><br>Have you done the following things to your wife in the last 12 months…		
501a (75)	Shouted or yelled at her?	Often…………………………………..………….1<br>Sometimes ………………………………..…….2<br>Never ……………………………………...….3<br>No response …………………………………..77	
501b (76)	Threatened to hurt her or someone she cares about?	Often…………………………………..………….1<br>Sometimes ………………………………..…….2<br>Never ……………………………………...….3<br>No response …………………………………..77	
501c (77)	Pushed her, shaken her, or thrown something at her?	Often…………………………………..………….1<br>Sometimes ………………………………..…….2<br>Never ……………………………………...….3<br>No response …………………………………..77	

501d (78)	Slapped her or twisted her arm.	Often……………………………………..………..1<br>Sometimes …………………………………..……..2<br>Never ……………………………………….....…3<br>No response ………………………………….77	
501e (79)	Punched her with your fist or with something else that could hurt her.	Often……………………………………..………..1<br>Sometimes …………………………………..……..2<br>Never ……………………………………….....…3<br>No response ………………………………….77	
	Within the last 12 months …..		
502a (80)	Have you physically forced your wife/partner to have sexual intercourse when she expressed that she not want to?	Often……………………………………..………..1<br>Sometimes …………………………………..……..2<br>Never ……………………………………….....…3<br>No response ………………………………….77	
502b (81)	Have you yelled or used physical force against your wife/partner to discourage her from using a modern method of family planning?	Often……………………………………..………..1<br>Sometimes …………………………………..……..2<br>Never ……………………………………….....…3<br>No response ………………………………….77	
**Justification of Intimate Partner Violence**			
(82)	Sometimes a husband is annoyed or angered by things that his wife does. In your opinion is a husband justified in hitting or beating his wife if she…		
503a (83)	Goes out without telling him?	Yes ……………………………………………….1<br>No …………………………………………….….2<br>No response ………………………………….77	
503b (84)	Neglects the children?	Yes ……………………………………………….1<br>No …………………………………………….….2<br>No response ………………………………….77	
503c (85)	Argues with him?	Yes ……………………………………………….1<br>No …………………………………………….….2<br>No response ………………………………….77	
503d (86)	Refuses to have sex with him?	Yes ……………………………………………….1<br>No …………………………………………….….2<br>No response ………………………………….77	
503e (87)	Burns the food?	Yes ……………………………………………….1<br>No …………………………………………….….2<br>No response ………………………………….77	
503f (88)	Uses a modern method of family planning without his permission	Yes ……………………………………….……..1<br>No ………………………………………..…..2<br>No response ……………………………..….77	
(89)	*Now I want to ask you a few questions about when you were a child. Think about the time before you were 15 years old and living with parents or another caretaker.*		
504 (90)	Did you see or hear your mother or other women in your home being beaten by your father or another man living in the home?	Never……………………………………………1<br>Sometimes……………………..………………2<br>Often………………………….………………3	
505 (91)	Were you threatened with physical punishment in your home?	Never……………………………………………1<br>Sometimes……………………..………………2<br>Often………………………….………………3	
506 (92)	Did your parents, or caretakers, or any other adults in the home slap or beat you?	Never……………………………………………1<br>Sometimes……………………..………………2<br>Often………………………….………………3	

SECTION 6: Attitudes and Norms related to Intimate Partner Violence			
**Intimate Partner Violence: Outcome Expectation**			
(93)	*I'm now going to read you a series of statements regarding violence by a husband against his wife, and then I will ask you if you strongly agree, agree, disagree or strongly disagree on each of them.*		
601 (94)	A husband beats his wife to correct her bad behavior.	Strongly agree……………………………………1<br>Agree....…………………………………………2<br>Disagree………………………………………..3<br>Strongly disagree………………………………4	
602 (95)	A husband beating his wife is a normal part of married life.	Strongly agree……………………………………1<br>Agree....……………………….………………….2<br>Disagree………………………………………..3<br>Strongly disagree………………………………4	
603 (96)	If the neighbors see or hear a husband beating his wife, they will try to stop him	Strongly agree……………………………………1<br>Agree....……………………….………………….2<br>Disagree………………………………………..3<br>Strongly disagree………………………………4	
**Intimate Partner Violence: Attitudes, Perceived Control and Intentions**			
(97)	*I want to read to you a few more statements. Please tell me how much you agree with each statement. Sometimes I will ask you for slightly different answers but I will tell you when we get to those statements.*		
604 (98)	According to the scripture a husband is supposed to discipline his wife.	Strongly agree……………………………………1<br>Agree....……………………….………………….2<br>Disagree………………………………………..3<br>Strongly disagree………………………………4	
605 (99)	Giving equal weight to what my wife (partner) says in making decisions is__________to me.	Extremely important…………………………..….1<br>Important…………………….…………………….2<br>Unimportant…………………………………….....3<br>Extremely unimportant………………………..….4	
606 (100)	If a man does not beat his wife, people will think he is not manly.	Strongly agree……………………………………1<br>Agree....……………………….………………….2<br>Disagree…………………………...…………..3<br>Strongly disagree………………………………4	
607 (101)	I would use other nonviolent strategies to manage conflict with my husband (partner) if I knew them.	Strongly agree……………………………………1<br>Agree....……………………….………………….2<br>Disagree………………………………………..3<br>Strongly disagree………………………………4	

Intimate Partner Violence: Injunctive Norms			
(102)	For this next set of questions, I will read a series of statements. For each statement, I want to know what you think people expect others to do. You can say strongly agree, agree, disagree or strongly disagree.		
608 (103)	People in this congregation expect a husband to force his wife to have sex even when she does not want to	Strongly agree…………………………………..1<br>Agree……………………………………………2<br>Disagree…………………………………………3<br>Strongly disagree………………………………4	
609 (104)	People in this congregation think it is ok for a husband to beat his wife at times	Strongly agree…………………………………..1<br>Agree……………………………………………2<br>Disagree…………………………………………3<br>Strongly disagree………………………………4	
610 (105)	Faith leaders think it is ok for a husband to beat his wife at times	Strongly agree…………………………………..1<br>Agree……………………………………………2<br>Disagree…………………………………………3<br>Strongly disagree………………………………4	
611 (106)	Faith leaders think it is ok for a husband to force his wife to have sex even when she does not want to	Strongly agree…………………………………..1<br>Agree……………………………………………2<br>Disagree…………………………………………3<br>Strongly disagree………………………………4	
612 (107)	It is appropriate for a husband to beat his wife at times	Strongly agree…………………………………..1<br>Agree……………………………………………2<br>Disagree…………………………………………3<br>Strongly disagree………………………………4	
**Intimate Partner Violence: Subjective Norms**			
613 (108)	For matters related to my relationship with my wife, whose opinion matters to you?	Wife……..…………………………………… 1<br>Friends…………………………………………2<br>Mother…………………………………………3<br>Father…………………………………………..4<br>Mother-in-law…………………………………..5<br>Father-in-law…………………………………..6<br>Faith leader……………………………………7<br>Sister……………………………………………8<br>Brother…………………………………………9<br>Other female relative…………………………10<br>Other male relative……………………………11<br>Other……………………………………………88<br>Specify_______________________________	

614 (109)	My wife thinks it is ok for me to beat her at times.	Strongly agree……………………………………1<br>Agree……………………………………………2<br>Disagree…………………………………………3<br>Strongly disagree………………………………4	
615 (110)	My wife thinks it is ok for me to force her to have sex even when she does not want to.		
616 (111)	Faith leaders in this congregations think it is ok for me to beat my wife at times	Extremely likely……………………………………1<br>Likely………………………………………………2<br>Unlikely………………………………………………3<br>Extremely unlikely……………………………4	
617 (112)	People whose opinion is important to me think it is ok for me to beat my wife at times.	Strongly agree……………………………………1<br>Agree……………………………………………2<br>Disagree…………………………………………3<br>Strongly disagree………………………………4	
**Intimate Partner Violence: Motivation to Comply**			
618 (113)	In matters of my relationship with my wife, I want to do what my wife wants me to do.		
619 (114)	In matters of my relationship with my wife, I want to do what my faith leaders in this congregation want me to do.	Strongly agree……………………………………1<br>Agree……………………………………………2<br>Disagree…………………………………………3<br>Strongly disagree………………………………4	
**Intimate Partner Violence: Descriptive Norms**			
(115)	*Now I would like to know what people in your congregation actually do. Think about what is normal or typical behavior when you respond to these statements. Do you think that these statements are true for most, many, some or none of the newly married couples or first time parents in this congregation?*		
620 (116)	A husband beats his wife	None……………………………………………1<br>Some……………………………………………2<br>Many……………………………………………3<br>Most……………………………………………4	
621 (117)	A husband forces his wife to have sex even when she does not want to	None……………………………………………1<br>Some……………………………………………2<br>Many……………………………………………3<br>Most……………………………………………4	
**SECTION 7: Gender Roles and Positive Masculinities**			

Gender and Postive Masculiniities: Attitudes, Injunctive Norms and Intentions			
(118)	*I want to read to you a few more statements. Please tell me whether you how much you agree with the statements.*		
701<br>(119)	I give equal weight to what my wife says when making a decision	Strongly agree……………………………….1<br>Agree…..…………………….………………..2<br>Disagree……………………………………..3<br>Strongly disagree……………………………4	
702<br>(120)	People whose opinions are important to me, approve of the husband sharing in the household work such as doing dishes, cleaning and cooking.	Strongly agree……………………………….1<br>Agree…..…………………….………………..2<br>Disagree……………………………………..3<br>Strongly disagree……………………………4	
703<br>(121)	I believe men and women were created equal.	Strongly agree……………………………….1<br>Agree…..…………………….………………..2<br>Disagree……………………………………..3<br>Strongly disagree……………………………4	
704<br>(122)	My wife can express her opinion even if I disagree.	Strongly agree……………………………….1<br>Agree…..…………………….………………..2<br>Disagree……………………………………..3<br>Strongly disagree……………………………4	
705<br>(123)	People whose opinions are important to me, approve of the husband sharing in the responsibilities of child care	Strongly agree……………………………….1<br>Agree…..…………………….………………..2<br>Disagree……………………………………..3<br>Strongly disagree……………………………4	
706<br>(124)	It is important to me that I play a role in bringing up our children beyond being the financial provider.	Strongly agree……………………………….1<br>Agree…..…………………….………………..2<br>Disagree……………………………………..3<br>Strongly disagree……………………………4	
707<br>(125)	Most newly married couples and first time parents that I know in this congregation approve of the husband sharing in the household work such as washing dishes, cleaning and cooking	Strongly agree……………………………….1<br>Agree…..…………………….………………..2<br>Disagree……………………………………..3<br>Strongly disagree……………………………4	
708<br>(126)	Most newly married couples and first time parents that I know in this congregation approve of the husband sharing in the responsibilities of child care.	Strongly agree……………………………….1<br>Agree…..…………………….………………..2<br>Disagree……………………………………..3<br>Strongly disagree……………………………4	
Gender and Positive Masculinities: Subjective Norms			

709 (127)	My wife (partner) thinks we should both share in the house work such as doing dishes, cleaning and cooking.	Strongly agree…………………………………1<br>Agree....…………………….………………….2<br>Disagree………………………………………..3<br>Strongly disagree………………………………4	
710 (128)	My wife (partner) thinks we should both share in the responsibility of child care.	Strongly agree…………………………………1<br>Agree....…………………….………………….2<br>Disagree………………………………………..3<br>Strongly disagree………………………………4	
711 (129)	Faith leaders in this congregation think we should both share in the responsibility of child care.	Strongly agree……………………………………1<br>Agree....…………………….…………………….2<br>Disagree…………………………………………..3<br>Strongly disagree…………………………………4	
717 (130)	Faith leaders in this congregation think we should both share in the house work such as doing dishes, cleaning and cooking.	Strongly agree……………………………………1<br>Agree....…………………….…………………….2<br>Disagree…………………………………………..3<br>Strongly disagree…………………………………4	
**Gender and Positive Masculinities: Descriptive Norms**			
(131)	*Now I would like to know what people in your congregation actually do. Think about what is normal or typical behavior when you respond to these statements. Do you think that these statements are true for most, many, some or none of the people in this congregation?*		
713 (132)	The husband shares in the work around the house such as doing dishes, cleaning and cooking.	None ……………………………….................1<br>Some ……………..……………………..……….2<br>Many ………………..…………………….……..3<br>Most ……………………..…………………..……..4	
714 (133)	The husband shares in the responsibilities of child care.	None ……………………………….................1<br>Some ……………..……………………..……….2<br>Many ………………..…………………….……..3<br>Most ……………………..…………………..……..4	
**SECTION 8: Relationship Quality**			
(134)	*Now I want to ask you a few questions about your relationship with your wife (partner).*		
801 (135)	In the last month, did you tell your wife that you appreciated her?	Yes………………………………………………1<br>No ……………………………………………..2	
802 (136)	In the past month, did you take time to listen to your wife's concerns?	Yes……………………………………………..1<br>No ……………………………………………..2	
803 (137)	In the past month, did you and your wife talk about things that frustrated you?	Yes………………………………………………1<br>No ………………………………………………2	

804 (138)	In the past month, did you and your wife talk about things that made you happy?	Yes……………………………………………..1<br>No ……………………………………………..2	

Thank you for participating in this survey!
