# Supplementary material for: Getting to intent: Are social norms influencing intentions to use modern contraception in the DRC?
Source: PLoS One. 2019 Jul 16;14(7):e0219617. doi: 10.1371/journal.pone.0219617 (PMC6634398; doi:10.1371/journal.pone.0219617)
Supplement: S1 Table — (DOCX) [file pone.0219617.s003.docx]

**S1 Table. Descriptive statistics of items included in exploratory factor analysis**

| Item Text | Observations | Response Options | Mean |
| --- | --- | --- | --- |
| Family Planning (FP) Norms |  |  |  |
| If I use a modern method of FP it would not be against my religion. | 900 | 1=Strongly disagree 2=Disagree 3=Agree 4=Strongly agree | 2.52 |
| If I use a modern method of FP I will get a reputation for being promiscuous. | 899 | 1=Strongly agree 2=Agree 3=Disagree 4=Strongly disagree | 2.91 |
| Members of this congregation think it is appropriate for NMCs to use modern methods of FP. | 898 | 1=Strongly disagree 2=Disagree 3=Agree 4=Strongly agree | 2.30 |
| Members of this congregation think it is appropriate for FTPs to use modern methods of FP. | 900 | 1=Strongly disagree 2=Disagree 3=Agree 4=Strongly agree | 2.04 |
| Faith leaders in this congregation think it is appropriate for FTPs to use a modern method of FP. | 898 | 1=Strongly disagree 2=Disagree 3=Agree 4=Strongly agree | 2.05 |
| Faith leaders think it is appropriate for NMCs to use a modern method of FP. | 897 | 1=Strongly disagree,  2=Disagree  3=Agree  4=Strongly agree | 2.33 |
| In matters related to FP, people whose opinions are important to me think I should use a modern method of FP. | 884 | 1=Strongly disagree 2=Disagree 3=Agree 4=Strongly agree | 1.99 |
| My partner thinks that we, as a couple, should use a modern method of FP. | 896 | 1=Strongly disagree 2=Disagree 3=Agree 4=Strongly agree | 2.00 |
| Faith leaders in this congregation think my partner and I should use a modern method of FP. | 900 | 1=Strongly disagree 2=Disagree 3=Agree 4=Strongly agree | 2.15 |
| In matters related to FP, how important is it for you to do what the faith leaders in this congregation want you to do? | 899 | 1=Not at all important 2=Not so important 3=Important 4=Very important | 2.14 |
| In matters related to FP, how important is it for you to do what people whose opinion matters to you want you to do? | 900 | 1=Not at all important  2=Not so important  3=Important  4=Very important | 2.18 |
| Perceived proportion of congregation in which NMCs use a modern method of FP | 899 | 1=None, 2=Some, 3=Many, 4=Most | 1.98 |
| Perceived proportion of congregation in which FTPs use a modern method of FP | 898 | 1=None, 2=Some, 3=Many, 4=Most | 2.08 |
| Household Gender Equity Norms |  |  |  |
| People whose opinions are important to me approve of the husband sharing in the household work. | 898 | 1=Strongly disagree 2=Disagree 3=Agree 4=Strongly agree | 2.30 |
| People whose opinions are important to me approve of husbands sharing in the responsibilities of child care. | 897 | 1=Strongly disagree 2=Disagree 3=Agree 4=Strongly agree | 1.94 |
| Most newly married couples and first-time parents that I know in this congregation approve of the husband sharing in the household work. | 899 | 1=Strongly disagree 2=Disagree 3=Agree 4=Strongly agree | 2.34 |
| Most newly married couples and first-time parents that I know in this congregation approve of the husband sharing in the responsibilities of child care. | 899 | 1=Strongly disagree  2=Disagree  3=Agree  4=Strongly agree | 1.92 |
| My partner thinks we should both share in the housework. | 898 | 1=Strongly disagree 2=Disagree 3=Agree 4=Strongly agree | 2.40 |
| My partner thinks we should both share in the responsibility of child care. | 896 | 1=Strongly disagree 2=Disagree 3=Agree 4=Strongly agree | 1.93 |
| Faith leaders in this congregation think my partner and I should both share in the responsibility of child care. | 900 | 1=Strongly disagree 2=Disagree 3=Agree 4=Strongly agree | 1.93 |
| Faith leaders in this congregation think my partner and I should both share in the housework. | 900 | 1=Strongly disagree 2=Disagree 3=Agree 4=Strongly agree | 2.39 |
| Perceived proportion of congregation in which the husband shares in the work around the house | 900 | 1=None, 2=Some, 3=Many, 4=Most | 1.80 |
| Perceived proportion of congregation in which the husband shares in the responsibilities of child care | 898 | 1=None, 2=Some,  3=Many, 4=Most | 2.32 |
