# Supplementary material for: Getting to intent: Are social norms influencing intentions to use modern contraception in the DRC?
Source: PLoS One. 2019 Jul 16;14(7):e0219617. doi: 10.1371/journal.pone.0219617 (PMC6634398; doi:10.1371/journal.pone.0219617)
Supplement: S2 Table — (DOCX) [file pone.0219617.s004.docx]

**S2 Table. Descriptive statistics of latent constructs in structural equation models**

| Latent Construct/Derived Variable | Observations | Mean | Min | Max |
| --- | --- | --- | --- | --- |
| Family Planning Norm – Faith Community & Reference Group Approval of FP Use | 711 | 0.44 | -1.34 | 2.10 |
| Family Planning Norm – Perception of Prevalence of FP Use in Congregation | 711 | 0.57 | -0.94 | 2.52 |
| Household Gender Role Norm – Role in Chores | 711 | 0.13 | -2.38 | 1.98 |
| Household Gender Role Norm – Role in Child Care | 711 | 0.12 | -2.19 | 1.85 |
| Relationship Quality | 711 | 0.58 | -0.97 | 1.12 |
| Couple Communication | 711 | 0.64 | -0.46 | 1.31 |
