# Supplementary material for: Getting to intent: Are social norms influencing intentions to use modern contraception in the DRC?
Source: PLoS One. 2019 Jul 16;14(7):e0219617. doi: 10.1371/journal.pone.0219617 (PMC6634398; doi:10.1371/journal.pone.0219617)
Supplement: S3 Table — (DOCX) [file pone.0219617.s005.docx]

**S3 Table. Total effects of social norm constructs on intention to use modern family planning, separated by direct effects of social norms on outcome and indirect effects via mediation of couple’s relationship quality and communication**

|  | Women | | |  | Men | | |
| --- | --- | --- | --- | --- | --- | --- | --- |
|  | Effect Estimate (Standard Error) | | |  | Effect Estimate (Standard Error) | | |
|  | Direct | Indirect | Total |  | Direct | Indirect | Total |
| FP Norms |  |  |  |  |  |  |  |
| Faith Community & Reference Group Approval of FP Use | 0.596***  (0.090) | -0.01  (0.014) | 0.586***  (0.089) |  | -0.038  (0.452) | 0.396  (0.448) | 0.358***  (0.067) |
| Perception of Prevalence of FP Use in Congregation | 0.048  (0.069) | 0.021  (0.029) | 0.069  (0.065) |  | 0.474***  (0.179) | -0.072  (0.180) | 0.402***  (0.059) |
| Household Gender Equity Norms |  |  |  |  |  |  |  |
| Role in Chores | -0.073  (0.103) | -0.002  (0.018) | -0.075  (0.104) |  | -0.670  (0.223) | 0.039  (0.219) | -0.328***  (0.079) |
| Role in Child Care | 0.252*  (0.126) | 0.032  (0.034) | 0.284*  (0.119) |  | 0.181  (0.376) | 0.257  (0.367) | 0.438***  (0.093) |
